# Splicing factor SRSF1 promotes breast cancer progression via oncogenic splice switching of PTPMT1

**DOI:** 10.1186/s13046-021-01978-8

**Published:** 2021-05-15

**Authors:** Jun-Xian Du, Yi-Hong Luo, Si-Jia Zhang, Biao Wang, Cong Chen, Gui-Qi Zhu, Ping Zhu, Cheng-Zhe Cai, Jing-Lei Wan, Jia-Liang Cai, Shi-Ping Chen, Zhi Dai, Wei Zhu

**Affiliations:** 1 Department of General Surgery, Zhongshan Hospital, Fudan University, Shanghai, 200032 China; 2 Liver Cancer Institute, Zhongshan Hospital, Fudan University & State Key Laboratory of Genetic Engineering, Fudan University, Shanghai, 200032 China; 3 Key Laboratory of Carcinogenesis and Cancer Invasion, Fudan University, Ministry of Education, Shanghai, 200032 China; 4 Department of Physiology and Pathophysiology, School of Basic Medical Sciences, Fudan University, No. 130 Dongan Road, Shanghai, 200032 China

**Keywords:** Breast cancer, Alternative splicing, SRSF1, PTPMT1, P-AKT-C-MYC

## Abstract

**Background:**

Intensive evidence has highlighted the effect of aberrant alternative splicing (AS) events on cancer progression when triggered by dysregulation of the SR protein family. Nonetheless, the underlying mechanism in breast cancer (BRCA) remains elusive. Here we sought to explore the molecular function of SRSF1 and identify the key AS events regulated by SRSF1 in BRCA.

**Methods:**

We conducted a comprehensive analysis of the expression and clinical correlation of SRSF1 in BRCA based on the TCGA dataset, Metabric database and clinical tissue samples. Functional analysis of SRSF1 in BRCA was conducted in vitro and in vivo. SRSF1-mediated AS events and their binding motifs were identified by RNA-seq, RNA immunoprecipitation-PCR (RIP-PCR) and in vivo crosslinking followed by immunoprecipitation (CLIP), which was further validated by the minigene reporter assay. PTPMT1 exon 3 (E3) AS was identified to partially mediate the oncogenic role of SRSF1 by the P-AKT/C-MYC axis. Finally, the expression and clinical significance of these AS events were validated in clinical samples and using the TCGA database.

**Results:**

SRSF1 expression was consistently upregulated in BRCA samples, positively associated with tumor grade and the Ki-67 index, and correlated with poor prognosis in a hormone receptor-positive (HR+) cohort, which facilitated proliferation, cell migration and inhibited apoptosis in vitro and in vivo. We identified SRSF1-mediated AS events and discovered the SRSF1 binding motif in the regulation of splice switching of PTPMT1. Furthermore, PTPMT1 splice switching was regulated by SRSF1 by binding directly to its motif in E3 which partially mediated the oncogenic role of SRSF1 by the AKT/C-MYC axis. Additionally, PTPMT1 splice switching was validated in tissue samples of BRCA patients and using the TCGA database. The high-risk group, identified by AS of PTPMT1 and expression of SRSF1, possessed poorer prognosis in the stage I/II TCGA BRCA cohort.

**Conclusions:**

SRSF1 exerts oncogenic roles in BRCA partially by regulating the AS of PTPMT1, which could be a therapeutic target candidate in BRCA and a prognostic factor in HR+ BRCA patient.

**Supplementary Information:**

The online version contains supplementary material available at 10.1186/s13046-021-01978-8.

## Background

Breast cancer (BRCA) is one of the most prevalent women cancers in women, worldwide and seriously endangers the lives and health of women. According to cancer statistics in 2019, BRCA ranks highest in cancer morbidity and mortality [1]. Molecular subtype classifications have enhanced our understanding of BRCA and can guide BRCA treatments. Statistics in 2019 showed that among all age groups, HR+ BRCA is the most prevalent molecular subtype [2]. It is necessary to explore the pathogenesis of BRCA to find possible therapeutic targets.

More than 95% of all human genes undergo alternative splicing (AS) after transcription, which enriches the diversity of transcripts and proteins [3]. AS patterns are divided into 8 types, skipped exon (SE), intron retention (IR), mutually exclusive exon (MXE), selection use of alternative 5′ or 3′ splice site (A5SS/A3SS), alternative 3′ untranslated regions (UTRs) and alternative first or last exon (AFE/ALE) [4], among which SE is the most common AS pattern. As a widespread and pivotal posttranscriptional regulation method, aberrant AS events are involved in the pathogenesis of various diseases, including cancer. Switching of splice isoforms caused by abnormal AS is widely involved in the regulation of cancer phenotypes, including proliferation, apoptosis, cell cycle progression, invasion and metastasis, angiogenesis, abnormal energy metabolism and immune escape [5]. In BRCA, multiple genes such as BRAC1, FGFR1/2, HER2, and DMTF1/KLF6 have been reported to undergo abnormal AS, resulting in isoforms with different or even opposite functions [6].

AS, a highly dynamic and complex process, is regulated by the spliceosome, which is composed of 5 small nuclear ribonucleic acids (snRNA) and hundreds of protein factors. Each snRNA is combined with various proteins to form a stable small nuclear ribonucleoprotein (snRNP). With the assistance of other splicing factors (SFs), the spliceosome completes the assembly, activation, catalysis and dissociation [7]. SR proteins belong to an important SF family. The common feature of classic SR proteins is that they all contain at least an RNA recognition motif (RRM) and an Arg/Ser-rich (RS) domain [8]. The RRM domain is mainly responsible for the specific recognition and combination of the splicing regulatory elements (SREs) on the pre-mRNA, and the RS domain is commonly responsible for the assembly of the spliceosome. Moreover, the RS domain is enriched in arginine and serine, which can be posttranslationally modified [9].

Dysregulation of SFs is one of the main causes of hundreds of aberrant AS events in cancer [10–12], which influences oncogenesis and tumor progression, and sometimes even possesses prognostic value for cancer patients. SRSF1 is one of the fundamental SR proteins containing two RRMs and one RS domain. SRSF1 binds to different SREs to promote or inhibit splicing [13, 14]. In addition, SRSF1 exerts other biological functions, including transcriptional activation, RNA stabilization, mRNA transport, and translation control [15–18]. Strikingly, oncogenic roles of SRSF1 and the landscape of AS events regulated by SRSF1 have been observed in several cancers [19–23]. For instance, splice switching of MYO1B directly regulated by SRSF1 increases the oncogenic potential of glioma cells by the PDK1/AKT and PAK/LIMK pathways [22]. Notably a study has focused on the investigation of SRSF1-regulated AS targets in BRCA by analyzing the RNA-seq data of the human nontransformed mammary epithelial MCF10A cells [24]. Although the role of SRSF1 as an SF has been extensively investigated over the past two decades, our knowledge is limited about the clinical significance, specific targets and detailed regulatory mechanisms of SRSF1 in BRCA.

In this study, we sought to elucidate the expression, clinical correlation, biological function and underlying AS-regulating mechanism of SRSF1 in BRCA. Data obtained from public databases and clinical samples confirmed that SRSF1 was upregulated in BRCA, and positively associated with tumor grade and the Ki-67 index. SRSF1 also correlated with poor cancer prognosis in a hormone receptor-positive (HR+) cohort. Then, we identified AS events regulated by SRSF1 through RNA-seq, motif analysis, in vivo CLIP and minigene reporter assays, which demonstrated a position- and sequence-dependent modulation of AS by SRSF1 in BRCA. Then, we verified that SRSF1 promoted oncogenesis of BRCA cells partially by regulating PTPMT1 splice switching, and by further activating the P-AKT/C-MYC signaling pathway. Collectively, our study highlights the biological and clinical significance of SRSF1 in BRCA, including the SRSF1-dependent regulation of cancer-associated AS events, and investigates the underlying regulatory mechanisms in detail.

## Materials and methods

### Tissue samples and tissue microarray

Thirty-eight pairs of BRCA and corresponding adjacent tissues were obtained from BRCA patients undergoing surgery at Zhongshan Hospital after obtaining written informed consent. Fresh samples were quickly frozen at − 80 °C for qRT-PCR and RT-PCR assays. Tissue microarray chips composed of BRCA (*n* = 42) and normal (*n* = 25) tissues were used for immunohistochemistry (IHC) analysis.

### IHC and IHC score evaluation

IHC analysis was performed as previously described [25]. In short, sections were deparaffinized with xylene and then rehydrated, followed by antigenic retrieval. Sections were then treated with 3% peroxidase to remove endogenous peroxidase and prevent nonspecific background staining. Then, the sections were incubated with SRSF1 antibody, washed and incubated with secondary antibody. For a more detailed description of the IHC assay, see the supplemental materials.

After scanning with a PANNORAMIC panoramic slice scanner, Quant Center 2.1 software was used for evaluation. The H-score was calculated using the following formula: H-SCORE = ∑(PI×I), where PI represents the proportion of the positive signal pixel area; and I represents the color intensity [26]. The H-score was used to evaluate the protein expression levels of SRSF1 in tissue samples.

### Cell culture, reagents, siRNA, generation of lentivirus and construction of stable cell lines

MCF7, T47D and MDA-MB-231 (231) BRCA cell lines and human embryonic kidney 293 T cell lines were obtained from Dr. Feng Qiao at the Fudan University Shanghai Cancer Center. All cell lines were confirmed to lack mycoplasma contamination, but additional authentication was not conducted. DMEM (Gibco) containing 10% FBS (Gibco) was used to culture all cell lines in a humidified incubator at 37 °C with 5% CO_2_ [27, 28]. SRSF1 (sc-33,652) and Ki-67 (sc-23,900) antibodies from Santa Cruz were purchased for IHC staining and western blotting. GAPDH (ab8245) antibodies were purchased from Abcam and β-tubulin (M1305-2) antibodies were purchased from Hangzhou HuaAn Biotechnology Co.,Ltd. AKT1 (A17909), p85 PI3K (A4992) and S473 AKT1 (AP0637) antibodies were purchased from ABclonal. Additional details about these primary antibodies are summarized in Supplementary Table 1.

SiPTPMT-L was synthesized by Sangon Biotech and transfected into cells by using Lipofectamine™ 2000 Transfection Reagent based on the manufacturer’s instructions. The Sh-SRSF1/sh-PTPMT1-L plasmids were constructed with the pLKO.1 vector, and SRSF1/PTPMT1-L/PTPMT1/S overexpression plasmids were constructed with the pLenti-C-Myc-DDK-IRES-Puro vector. The inserted sequences are presented in Supplementary Table 2. The above mentioned target plasmids and empty vectors were separately cotransfected with the pM2.G and psPAX2 lentiviral packaging plasmids into 293 T cells to produce lentiviruses. Lentiviruses were mixed with 0.1% polybrene (4 μg/ml), cells were infected for 24 h, medium containing 5 μg/ml of puromycin was used to construct stable strains [28].

### qRT-PCR, RT-PCR, and western blotting

RNA extraction, reverse-transcription and PCR amplification were performed as previously described [25, 29]. Briefly, total RNA was extracted from cells, reverse transcribed into cDNA, and used as a template for PCR amplification by using qPCR SYBR Green Master Mix from YEASEN (11202ES03). The qRT-PCR primers are listed in Supplementary Table 3. RT-PCR was performed using AceTaq Master Mix (P412-01) from Vazyme for amplification according to the manufacturer’s instructions. Primers for RT-PCR were designed specifically to amplify two or more isoforms of different sizes (Supplementary Table 4). ACTB and 18S genes were used as internal controls for cells and tissues, respectively.

Western blotting was performed as previously described [25]. Equal amounts of protein extracts were separated by 10% SDS-PAGE, transferred to polyvinylidene difluoride membranes (Millipore, USA) and incubated with the indicated antibodies. GAPDH or β-tubulin antibodies were used as internal controls.

For more details about RT-PCR, qRT-PCR and western blotting methods, see the supplementary materials.

### Cell proliferation, colony formation, flow cytometry, wound-healing, Transwell and xenograft assays

Cell proliferation assays were conducted as previously described [30]. In brief, using a 96-well plate, zero-adjustment wells containing only culture medium. One hundred microliter cell suspensions containing 2000 of cells were added to each test well and incubated at 37 °C and 5% CO2 for 0, 24, 48, 72, 96 and 120 h. At each time point, 10 μl of Cell Counting Kit-8 (CCK-8) solution (Dojindo, Japan) was added, and the cells were incubated for 1.5 h in an incubator. Then, the absorbance at 450 nm was detected with an Infinite 200 spectrometer.

Colony formation assays [31] were performed in 6-well plates. For each cell type, 1000 cells and normal medium were added to each well, cultured in an incubator for 14 days, and fixed with 4% paraformaldehyde. The cells were then stained with crystal violet and imaged.

For the cell cycle assay [32], cells in 6-well plates were harvested and washed twice with prechilled PBS. Then, 70% ethanol was used to fix and dehydrate the cells overnight at 4 °C. After washing twice with PBS, the cells were resuspended in 150 μL of a solution containing 50 μg/mL of propidium iodide (PI), 0.1% Triton X-100 and 200 μg/mL of RNase A (Sigma-Aldrich, USA) and incubated at 37 °C for 30 min before analysis.

For the apoptosis assay [33], the Annexin V-FITC Apoptosis Detection Kit (BMS500FI-300, Thermo Fisher Scientific) was used. After 48 h of cultivation, cells and the medium were harvested and centrifuged. Then, Annexin V and PI were used to stain cells based on the manufacturer’s instructions. Then, all stained cells underwent flow cytometry analysis. Assays were performed in triplicate and were independently repeated three times.

The would-healing assay was performed as described [34]. For each cell type, an equal number of cells was plated in triplicate in 6-well dishes and inoculated to ensure that the fusion degree of the cells after attachment was 90%. A 10 μl pipette tip was used to scratch adherent cells, and the floating cells were washed with PBS that was then replaced with FBS-free medium. The cells were then imaged under a microscope. ImageJ was used to calculate the wounded area, which was recorded as W0. The cells were then returned to the incubator to continue culturing. Before each photo, the floating cells were washed with PBS and replaced with FBS-free medium. At specific time points, including 24 h, 48 h, and 72 h, the scars were photographed, and the wounded area was calculated and recorded as W1, W2, and W3. The relative migration area was calculated according to the following formula: (wounded area (W0-W1 or W2 or W3)/wounded area W0) × 100%.

Transwell assays were performed as described [34]. Using 24-well plates, 700 μl of medium containing 20% FBS was added to each well, and Transwell chambers with an 8 μm pore size were gently inserted into each well. Next, 200 μl of FBS-free medium containing 6^10^4^ cells was evenly added to each chamber. After 12 h of culture in a tissue culture incubator, MDA-MB-231 cells were removed, and MCF7 cells were removed after 72 h. A cotton swab was used to wipe off the cells that did not penetrate the upper layer of the chamber, which was then rinsed in PBS and fixed in 4% paraformaldehyde for 15 min, followed by staining in 0.1% crystal violet for 15 min. After washing thoroughly with PBS, migrated cells were imaged and quantified with ImageJ.

A xenograft tumor formation assay was performed by using 4-week-old female nude mice (BALB/c) that were obtained from the Shanghai Experimental Animal Research Center. The mice were subcutaneously injected in one of the flanks with the indicated cell types (T47D pLKO.1 or T47D shSRSF1, MDA-MB-231 pCMV or MDA-MB-231 PTPMT1-L or MDA-MB-231 PTPMT1-S) (5*10^6^ cells in 100 μl serum-free media containing 0.25 v/v Matrigel). Estradiol was used to promote the growth of tumors formed by T47D cells. All animals were maintained in pathogen-free conditions and in accordance with the guidelines of the Institutional Animal Care and Use Committee. Six weeks after injection with T47D cells and 3 weeks after injection with MDA-MB-231 cells, mice were euthanized, and tumors were extracted for further analysis [34]. The tumor volume was calculated using the following formula: tumor volume = length*width^2^/2 [28].

### Motif analysis of SRSF1-mediated alternative exons

According to the previously described CLIP analyses [35] and the characteristics of the SRSF1-binding sequence [22], we regarded GA-rich 6-mers that contained at least 1G and 1A with GA content ≥50% as potential SRSF1-binding sites. Potential SRSF1-binding motifs were analyzed according to previously described methods [36], and the Multiple EM for Motif Elicitation (MEME) suite was used to perform motif analysis [37]. For more details about motif analysis, see the supplementary materials.

### RNA immunoprecipitation-PCR (RIP-PCR) and in vivo crosslinking followed by immunoprecipitation (CLIP)

MCF7 cells cultured in 10 cm dishes were harvested, and RIP assays were carried out using SRSF1 (sc-33,652) antibodies and a Magna RIP kit (Millipore). The RIP assay was performed based on the instructions of the Magna RIP kit. RNA was extracted from the immunoprecipitate and reverse transcribed into cDNA for further RT-PCR detection. Primer sequences are listed in Supplementary Table 4.

An in vivo CLIP assay of RNA bound directly to SRSF1 was carried out as previously described with mild modifications [38]. Briefly, MCF7 cells were harvested, and ultraviolet cross-linking was conducted followed by immunoprecipitation using SRSF1 antibodies and a Magna RIP kit. Cell extracts were incubated with antibody-coated magnetic beads overnight at 4 °C followed by washing with wash buffer 3 times, and 100 μl of wash buffer was added. Then, the cells were incubated with RNaseT1 (100 U/μl) in 22 °C water for 1 h, followed by immediate incubation in an ice bath for 5 min. Beads were washed 3 times with wash buffer and then subjected to proteinase K digestion, RNA extraction and reverse transcription using random primers. RT-PCR assays were carried out with specifically designed primers to amplify skipped cassette exons and flanking exons. Primer sequences are listed in Supplementary Table 4.

### Minigene reporter assay

Minigenes were constructed as previously described [39]. Briefly, the PTPMT1-FL minigene was constructed by cloning a sequence consisting of exons 2-4 as well as 300 bp at each end of introns 2 and 3 into the PCDNA3.1 vector. Deletion mutant derivatives were designed based on the PTPMT1-FL minigene plasmid. The primers used for plasmid construction and exogenous PTPMT1 AS event detection are presented in Supplementary Tables 4 and 5, respectively. For more details about the minigene reporter assay, see the supplementary materials.

### RNA-seq and data analysis

Total RNA isolated from MCF7 cells transfected with control and shSRSF1 lentivirus was subjected to paired-end RNA-seq using an Illumina HiSeq 2000 system based on the manufacturer’s instructions. Read mapping and data analysis for differentially regulated AS events between two samples were carried out as previously described [36]. The raw sequence data have been submitted to the Gene Expression Omnibus with accession number GSE163025.

The sequencing data of MCF10A were obtained from a previous study [24], and the Venny 2.1.0 online tool (https://bioinfogp.cnb.csic.es/tools/venny/index.html) was used to plot the different and same genes involved in every type of AS event in MCF7 and MCF10A cells.

### Metabric and TCGA RNA-seq data analysis

The RNA expression and clinical information data in the Metabric dataset (*n* = 2509) were downloaded from cBioPortal (https://www.cbioportal.org). RNA-seq data (RPKM) from 1091 BRCA samples and 113 normal samples and their clinical characteristics were obtained from the TCGA database. The PSI (Percent-Spliced-In) of AS events was downloaded from TCGA SpliceSeq developed by MD Anderson Cancer Center (https://bioinformatics.mdanderson.org/public-software/tcgaspliceseq/). The correlation scatter plot between SRSF1 and Ki-67 was downloaded from the GEPIA database (http://gepia.cancer-pku.cn/).

### Statistical analysis

All data presented as histograms represent a mean value ± S.D. of the total number of independent experiments. Statistical analysis was performed by Student’s t-test at a significance level of *P* < 0.05. Survival curves were generated by Kaplan-Meier methods, with comparisons performed using the log-rank test. GraphPad Prism 7 and R software were used to visualize the results and analyze the statistical significance.

## Results

### SRSF1 is increased in BRCA, and its high expression predicts poor prognosis in HR+ BRCA patients

**Fig. 1:**
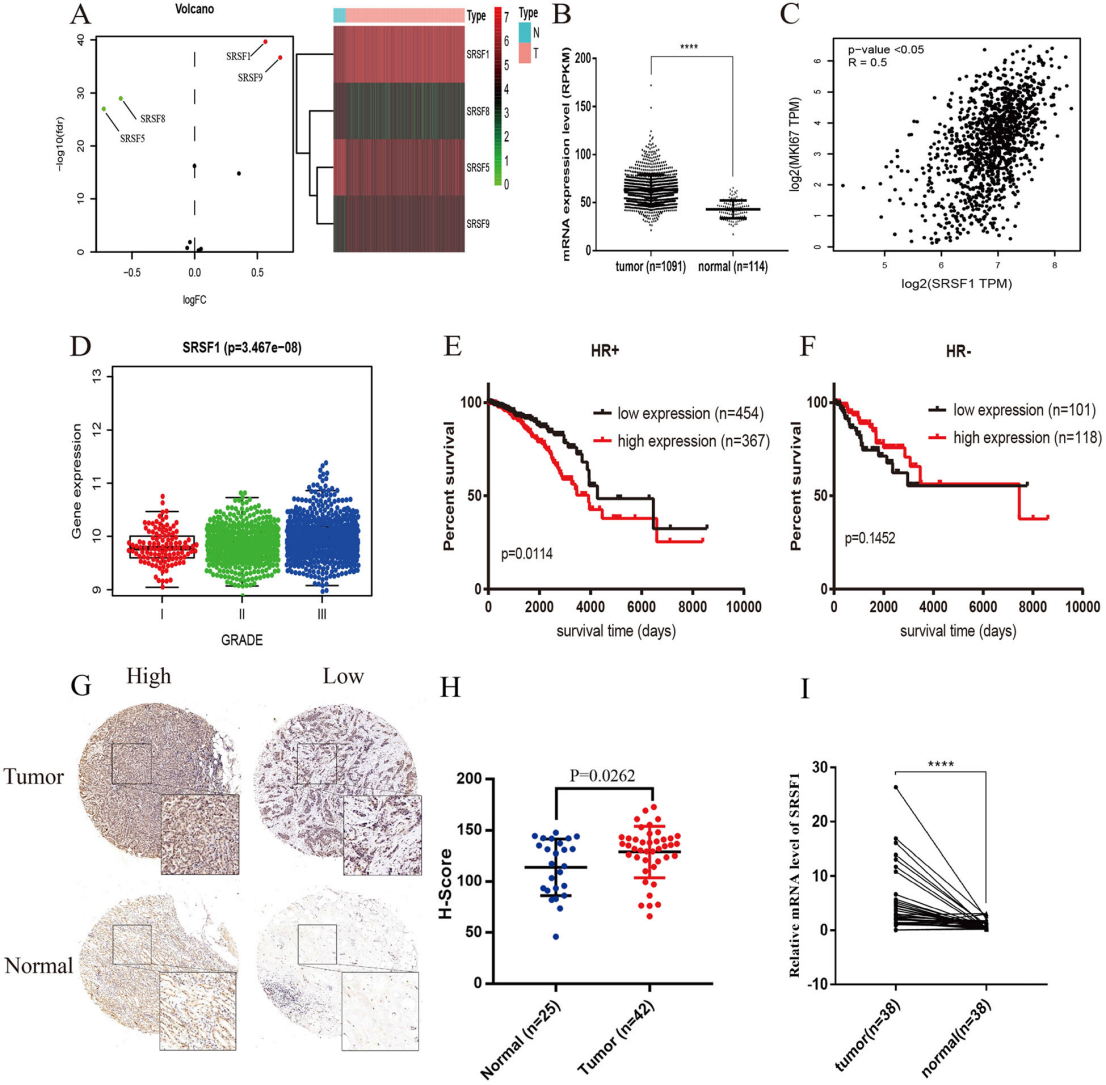
SRSF1 is upregulated in breast cancer samples and high expression of SRSF1 correlates with aggressive clinical characteristics and a poor prognosis in hormone receptor positive (HR+) subgroups. **a** The volcanic map and heatmap of differentially expressed SR family proteins in breast cancer samples (filtered criteria: |logFC| > =0.5, FDR < 0.05). **b** Scattered plots comparing SRSF1 mRNA expression levels in normal and tumor samples in TCGA dataset; Data are presented as mean ± SD; Statistical significance is calculated by unpaired t-test. **c** Correlation between relative expression of Ki67 and SRSF1 using Pearson correlation test based on TCGA dataset downloaded from GEPIA website. **d** Correlation between RNA expression levels of SRSF1 and histological grade based on Metabric dataset downloaded from cBioPortal using One-way ANOVA test. **e** Kaplan-Meier plots of ER/PR+ subgroup patients with high and low SRSF1 expression. **f** Kaplan-Meier plots of HR- subgroup patients with high and low SRSF1 expression. **g** Representative immunostaining images of SRSF1 in tumor and adjacent breast tissues (200*). **h** Scattered plots of SRSF1 protein expression assessed by blinded IHC analysis of tumor (*n* = 42) and adjacent breast tissues (*n* = 25). **i** Scattered plots of SRSF1 mRNA expression in tumor and paired adjacent normal tissues (*n* = 38;****, *P* < 0.0001; paired t-test)

To explore the expression alteration of SR family proteins in BRCA, we analyzed the differentially expressed SR proteins in the samples from the TCGA dataset (Fig. 1a). SRSF1/9 were upregulated, while SRSF5/8 were downregulated in BRCA samples (|logFC| > 0.5, FDR < 0.05). The scatter plot depicted that SRSF1 was significantly upregulated in BRCA tissue samples (*P* < 0.0001) (Fig. 1b). Additionally, the mRNA expression level of SRSF1 was positively correlated with the mRNA expression of Ki-67 (R = 0.5, *P <* 0.05) (Fig. 1c) and the tumor grade (*P* = 3.467e-08) (Fig. 1d). Furthermore, Kaplan-Meier analysis showed that patients with higher expression levels of SRSF1 had poorer overall survival (OS) in the HR+ cohort (*P* = 0.0114) (Fig. 1e), while there was no significant difference in the OS of the HR- cohort (*P* = 0.1452) (Fig. 1f). Based on the analysis results from public datasets, we further explored the SRSF1 expression difference in tumor and adjacent tissues. IHC and qRT-PCR assays confirmed that SRSF1 was upregulated in tumors compared with normal tissues at both the protein (*P* = 0.0262) and mRNA levels (*P* < 0.0001) (Fig. 1g-i). Taken together, these results strongly illustrate that SRSF1 is upregulated in BRCA samples and that SRSF1 upregulation is correlated with clinical severity and prognosis.

### SRSF1 plays oncogenic roles in BRCA in vitro and in vivo

**Fig. 2:**
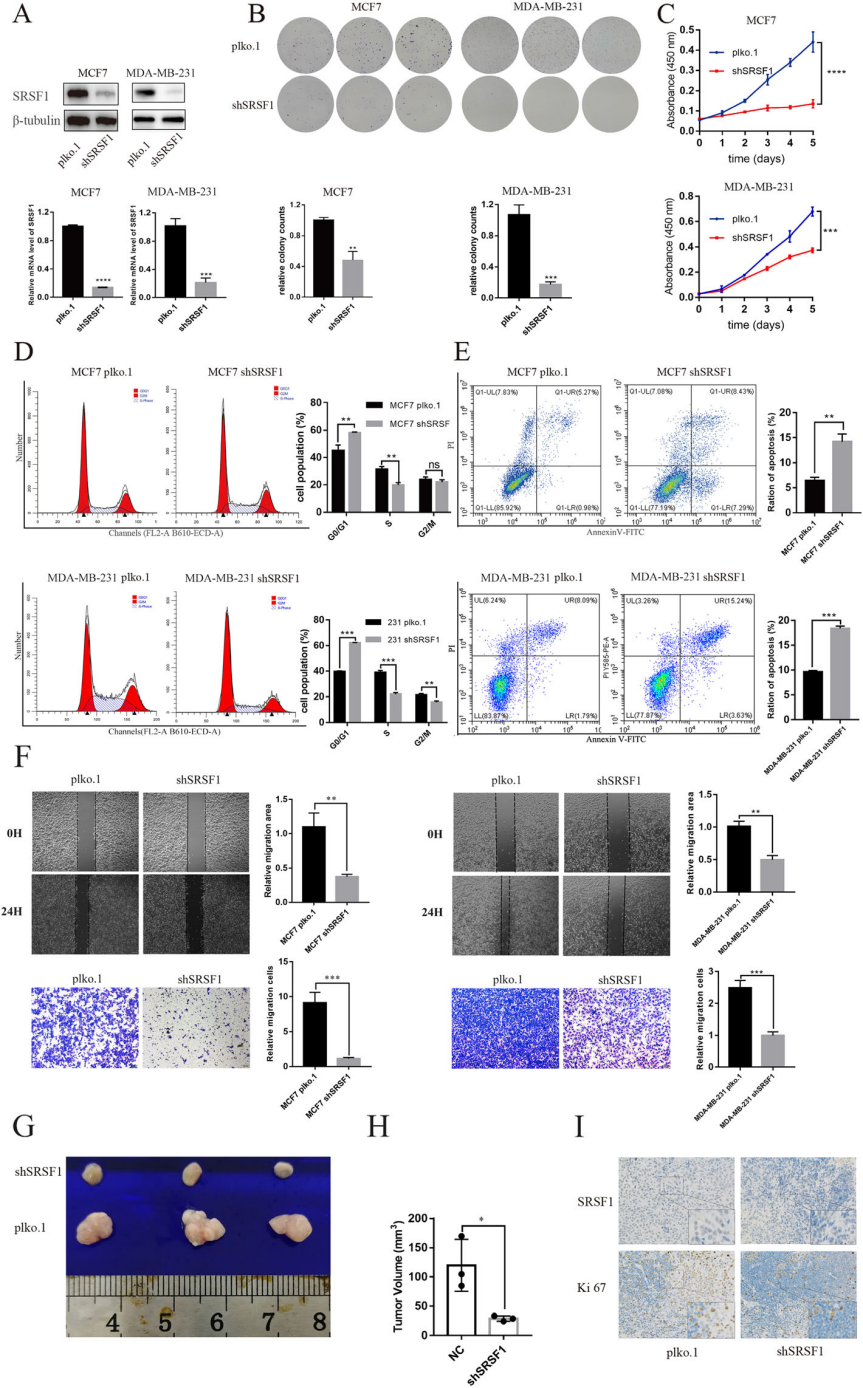
Knockdown of SRSF1 suppresses oncogenic roles in vitro and in vivo. **a** Luminal subtype cell line MCF7 or triple negative breast cancer cell line MDA-MB-231 are transfected with SRSF1 shRNA plasmid (shSRSF1) or control plasmid (pLKO.1). SRSF1 knockdown efficiency is confirmed by western blot and RT-qPCR. **b**, **c** Cell proliferation assay and clonogenic survival assay are performed using cells described in (**a**). **d** The cell cycle of cells described in (**a**) is analyzed by flow cytometry and the relative cell population of each cell cycle phase is quantified in the bar graph. **e** The ration of apoptosis in each group is calculated by flow cytometry. **f** Measurement of cell migration by wound-healing assays and transwell assays using cells described in (**a**). **g** T47D/sh-SRSF1 cells or control T47D/pLKO.1 cells are transplanted to nude mice. Tumors derived from T47D/sh-SRSF1 or control cells are excised and presented. **h** Volume of tumors are calculated and plotted. **i** Representative IHC results of SRSF1 and Ki67 in xenografts are presented (200*). *, *P* < 0.05; ** *P* < 0.01; *** *P* < 0.001; **** *P* < 0.0001

To fully investigate the biological function of SRSF1 in BRCA, we knocked down SRSF1 in MCF7, MDA-MB-231 and T47D cells. Western blotting and qRT-PCR analysis identified the knockdown efficiency of shSRSF1 lentivirus in MCF7, MDA-MB-231 and T47D cells (Fig. 2a, Supplementary Figure 1A). Knockdown of SRSF1 significantly inhibited cell proliferation, as confirmed by colony formation (Fig. 2b, Supplementary Figure 1B) and CCK-8 assays (Fig. 2c, Supplementary Figure 1C). Meanwhile, knockdown of SRSF1 caused G1-phase arrest (G1 phase, MCF7 pLKO.1 vs MCF7 shSRSF1, (44.96 ± 3.45)% vs (58.1 ± 0.40)%, *P* < 0.01; MDA-MB-231 pLKO.1 vs MDA-MB-231 shSRSF1, (39.71 ± 0.11)% vs (74.18 ± 3.25)%, *P* < 0.001; T47D pLKO.1 vs T47D shSRSF1, (72.43 ± 1.03)% vs (88.62 ± 0.85)%, *P* < 0.0001) (Fig. 2d, Supplementary Figure 1D) and promoted apoptosis (MCF7 pLKO.1 vs MCF7 shSRSF1, (6.42 ± 0.55)% vs (14.20 ± 1.25)%, *P* < 0.01; MDA-MB-231 pLKO.1 vs MDA-MB-231 shSRSF1, (9.62 ± 0.22)% vs (18.4 ± 0.39)%, *P* < 0.001; T47D pLKO.1 vs T47D shSRSF1, (11.77 ± 1.64)% vs (22.47 ± 3.61)%, *P* < 0.05) (Fig. 2e, Supplementary Figure 1E) in MCF7, MDA-MB-231 and T47D cells. Moreover, knockdown of SRSF1 inhibited the migration of MCF7 and MDA-MB-231 cells (Fig. 2f). To explore the tumorigenic effect of SRSF1, T47D cells transfected with shSRSF1 or control plasmid-constructed lentivirus were subcutaneously injected into the flanks of 4-week-old female nude mice. After 6 weeks of close monitoring, tumors were extracted for further analysis. In vivo analysis confirmed that knockdown of SRSF1 significantly inhibited tumorigenesis (Fig. 2g), as measured by tumor size (Fig. 2h). Furthermore, IHC staining demonstrated that knockdown of SRSF1 decreased the positive rate of the Ki-67 proliferation index (Fig. 2i). Collectively, these results show that knockdown of SRSF1 might dramatically promote cell cycle arrest and apoptosis, inhibit cell migration in vitro and inhibit BRCA proliferation in vivo and in vitro.

### Identification and validation of SRSF1-mediated AS events in BRCA cells

**Fig. 3:**
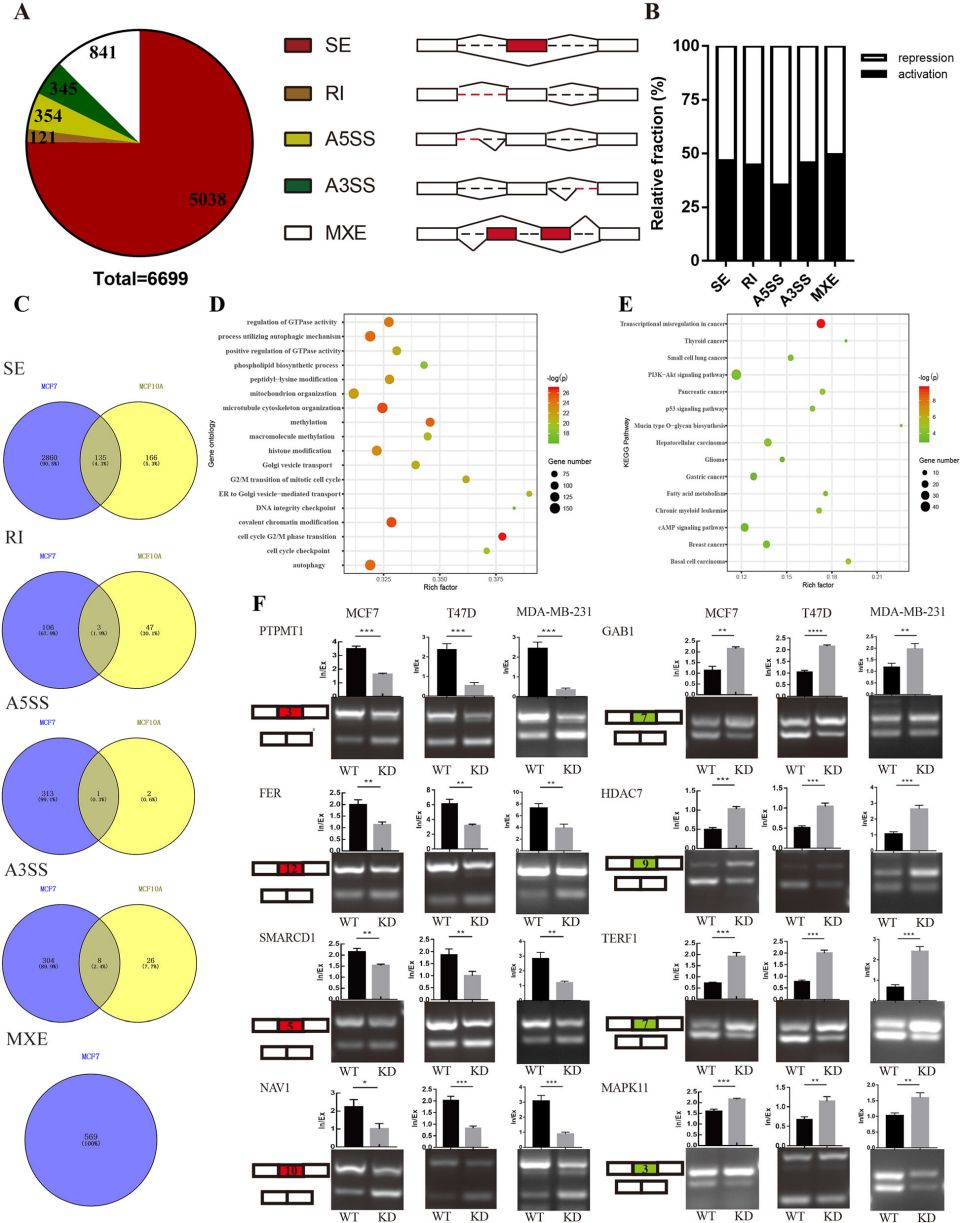
Alternative splicing (AS) and transcriptome profiles regulated by SRSF1 in breast cancer cells. **a** SRSF1-regulated AS events in MCF7 cell lines. The AS events are composed of 5 categories: skipped exon (SE), retained intron (RI), alternative 5′ splice site (A5SS), alternative 3′ splice site (A3SS), and mutually exclusive exon (MXE). **b** Relative fraction of AS events affected positively (activation) or negatively (repression) by SRSF1 in each category. **c** Overlapped AS-related genes in each category between MCF7 cells (our own RNA-seq data) and MCF10A cells (data referred from previous study). **d** Gene ontology of the differentially expressed genes between MCF7/shSRSF1 and MCF7/pLKO.1 cells. |LogFC| > 1, FDR < 0.05. **e** Enrichment KEGG pathway of differentially expressed genes. Rich factor = DEGs in this pathway term/ total annotated gene in this pathway term. **f** Representative SRSF1-affected SE events, RT-PCR results and quantification of their RNA products measured as inclusion/exclusion (In/Ex). Note that alternative exons for SRSF1-mediated inclusion are marked in red while SRSF1-mediated exclusion are marked in green. *, *P* < 0.05; ** *P* < 0.01; *** *P* < 0.001; **** *P* < 0.0001

Considering SRSF1 as a pivotal and classic SF, SRSF1-mediated AS events in human nontransformed mammary epithelial MCF10A cells were explored in a previous study [24]. However, the profile of SRSF1-regulated AS events in MCF7 breast cancer cells and the direct regulatory mechanism remain unclear. RNA-seq was performed on independent RNA samples extracted from SRSF1 gene knockdown and control MCF7 cells, and FDR < 0.05 and PSI differences between the knockdown and control group ≥0.1 were set as screening conditions. We identified 6699 splicing events, including 5038 skipped exons (SE), 121 retained introns (RI), 354 alternative 5′ splice sites (A5SS), 345 alternative 3′ splice sites (A3SS) and 841 mutually exclusive exons (MXE) (Fig. 3a). Subsequent analysis suggested that SRSF1 played a dual role in regulating AS, as it facilitated similar percentages of exon/intron inclusion (activation) and exclusion (repression) events (Fig. 3b). Next, we explored the similarities and differences between the genes involved in the AS events caused by knockdown of SRSF1 in MCF7 cells and the AS events caused by the overexpression of MCF10A in a previous study in distinct types of AS events. The results demonstrated that 135 common genes underwent SE events, 3 common genes underwent RI, 1 common gene underwent A5SS, and 8 common genes underwent A3SS. In addition, the MXE AS type was not involved in the previous study. Our sequencing results showed that after knocking down SRSF1 in MCF7 cells, 569 genes underwent 841 MXE events (Fig. 3c). Additionally, the biological functions of differentially expressed genes (DEGs) (|log2FC| > 1, FDR < 0.05) caused by knockdown of SRSF1 were investigated by GO and KEGG enrichment analysis. The results revealed that SRSF1-regulated DEGs were associated with autophagy, cell cycle checkpoints and histone modification (Fig. 3d). Moreover, DEGs were enriched in pivotal pathways, including transcriptional misregulation in cancer, PI3K/AKT signaling pathways and several cancer-related pathways (Fig. 3e). Next, to confirm the reliability of the RNA-seq data, we performed RT-PCR assays to validate the top 10 representative SRSF1-regulated abnormal SE events based on the FDR and the PSI differences between the SRSF1 knockdown and control groups. Detailed information on representative AS events was listed in Supplementary Table 6. Among them, SRSF1 promoted exon inclusion in PTPMT1, FER, SMARCD1 and NAV1 genes, while it facilitated exon exclusion in GAB1, HDAC7, TERF1 and MAPK11 genes (Fig. 3f). Moreover, overexpression of SRSF1 in 293 T cells mediated opposite splice switching trends of PTPMT1, SMARCD1, GAB1 and TERF1 genes compared to knockdown of SRSF1 in T47D cells (Supplementary Figure 2 A-B). It is worth noting that these key AS events were not identified in the previous study based on the MCF10A cells. These data indicate that SRSF1 strongly affects the biological functions of BRCA cells, especially in regulating AS.

### Motif analysis and in vivo CLIP assays reflect sequence- and position-dependent features of SRSF1 in regulating AS events

**Fig. 4:**
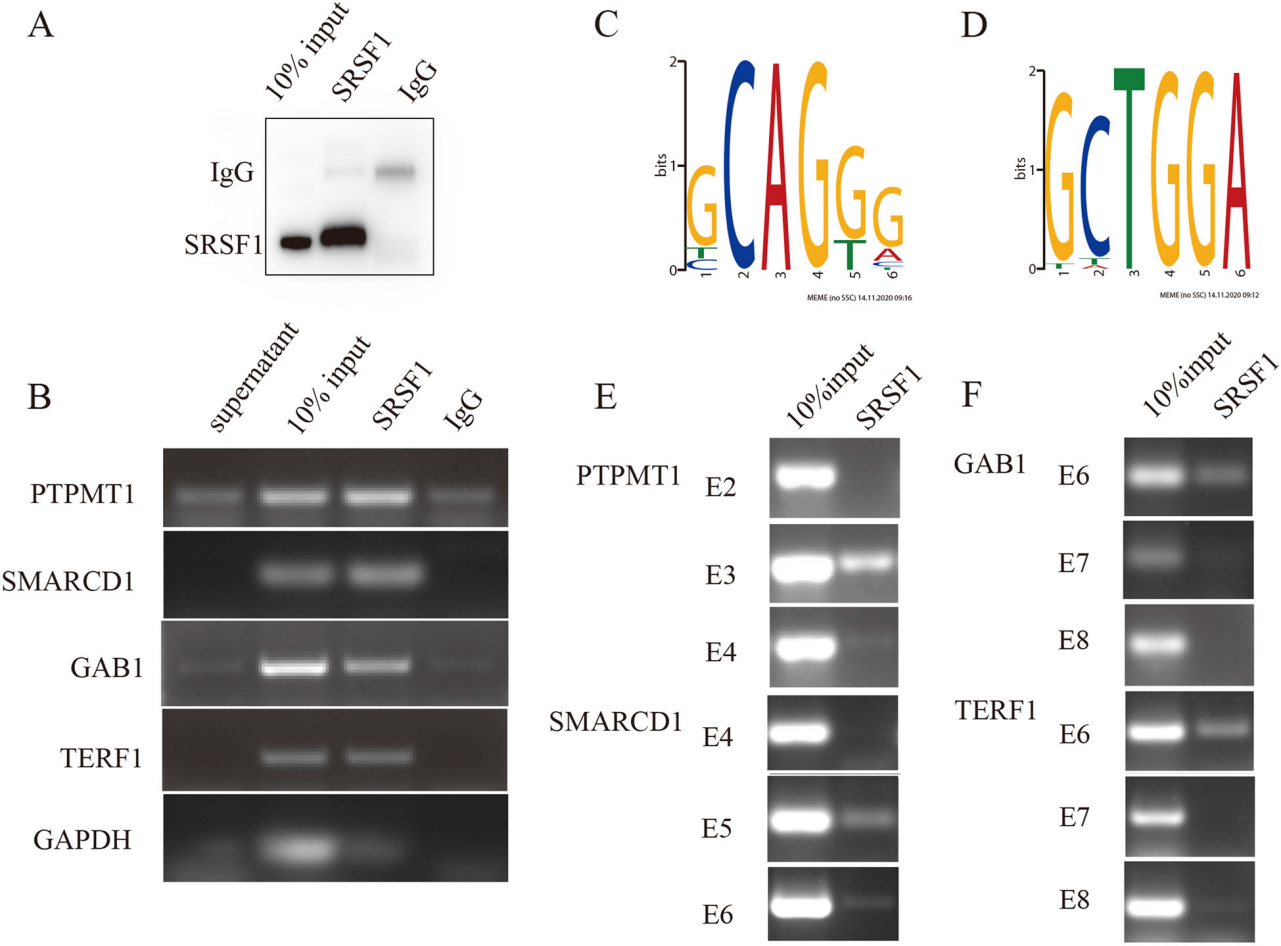
Motif analysis of SRSF1-affected AS events. **a** Western blot for testing the affinity of the SRSF1antibody to the SRSF1. **b** RIP-PCR results of representative SRSF1-regulated SE events. **c**, **d** Flowchart of the potential SRSF1 motifs derived from the 6-mers overrepresented in the activated (**c**) exon and the flanking constitutive exon of the repressed (**d**) cassette exon based on the RNA-seq data. **e**, **f** In vivo CLIP assay is performed and analyzed by RT-PCR with primer pairs complementary to SRSF1-affected cassette exons and two flanking constitutive exons

Moreover, RIP-PCR was conducted and confirmed that PTPMT1, SMARCD1, GAB1 and TERF1 transcripts were directly bound by SRSF1 (Fig. 4a-b). To identify the distribution of the SRSF1-binding motifs in SRSF1-activated or SRSF1-repressed SE events, we conducted de novo discovery of the SRSF1-binding motif utilizing the sequences of 100 (50 activated and 50 repressed) SRSF1-regulated SE events in MCF cells. AS of SRSF1-activated exons showed binding motifs within the cassette exons with a predominant enrichment of GCAGGG sequences (Fig. 4c). SRSF1-repressed exons preferred to bind the flanking constitutive exons with a predominant enrichment of GCTGGA sequences (Fig. 4d). Then, we tested whether SRSF1 bound to exons containing potential SRSF1-binding sequences in vivo. In vivo CLIP assays followed by RT-PCR analyses were performed with specific primer pairs. The results demonstrated that SRSF1 possessed predominant affinity within the cassette exons in SRSF1-activated AS events, including PTPMT1 and SMARCD1 (Fig. 4e). Meanwhile, SRSF1 preferred to bind the flanking constitutive exons in SRSF1-repressed AS events, such as GAB1 and TERF1 (Fig. 4f).

### Mechanistic insights into SRSF1-regulated PTPMT1 splice switching

**Fig. 5:**
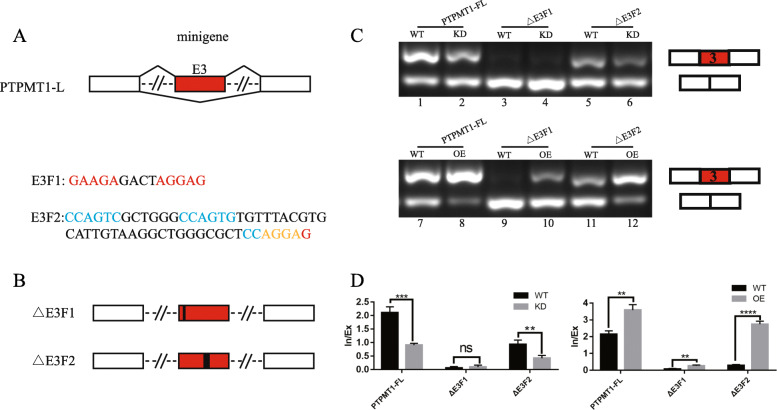
Mechanisms of SRSF1-regulated exon 3 of PTPMT1 inclusion. **a** Schematic representation of the PTPMT1 minigene construct. Two fragments (E3F1 and E3F2) each containing potential SRSF1 binding motifs are marked. Note that potential binding motifs of SRSF1 reported in article are marked in red, predicted motifs based on RNA-seq are marked in blue, and the overlap was marked in yellow. **b** Diagram of PTPMT1 minigene deletion mutant plasmids. **c** In vivo splicing analysis of PTPMT1 minigene and indicated deletion mutants in 293 T cells. **d** In/Ex index are shown at the bottom of the gel

To explore whether the binding of SRSF1 to PTPMT1 E3 is correlated with exon inclusion, first, a minigene reporter plasmid (PTPMT1-FL) composed of a genomic DNA fragment of PTPMT1 exons 2-4 and 300 bp sequences at each end of flanking constitutive introns was generated (Fig. 5a). Then, we carefully searched for the potential SRSF1 binding motifs based on previous reports (marked in red) and motif analysis predicted (marked in blue with the overlapping part marked in yellow) in PTPMT1 E3. We next explored the function of these motif elements in PTPMT1 E3 inclusion. We constructed a series of fragment deletion mutation plasmids based on the PTPMT1-FL minigene (Fig. 5b).

Fragment deletion mutation plasmids were then separately transfected into 293 T cells either alone or with shSRSF1/OE-SRSF1 plasmids, and the splicing pattern was analyzed by RT-PCR. The results revealed that the minigene could mimic the endogenous splicing pattern of PTPMT1. PTPMT1 E3 was predominantly included in control cells, whereas SRSF1 knockdown significantly inhibited the inclusion of PTPMT1 E3. However, SRSF1 overexpression increased the inclusion of PTPMT1 E3, indicating that the inclusion of PTPMT1 E3 was SRSF1 dependent (Fig. 5c, lanes 1-2 and 7-8). As depicted in Fig. 5c, the E3F1 deletion almost completely inhibited E3 inclusion and had either no response or a slight response to SRSF1 alteration (Fig. 5c, lanes 3-4 and 9-10). In contrast, the E3F2 deletion displayed little effect on splice switching and still responded to SRSF1 regulation (Fig. 5c, lanes 5-6 and 11-12). The RT-PCR results are quantified in histogram plots that are shown below the gel images (Fig. 5d).

### PTPMT1 splice switching promotes BRCA cell proliferation in vitro

**Fig. 6:**
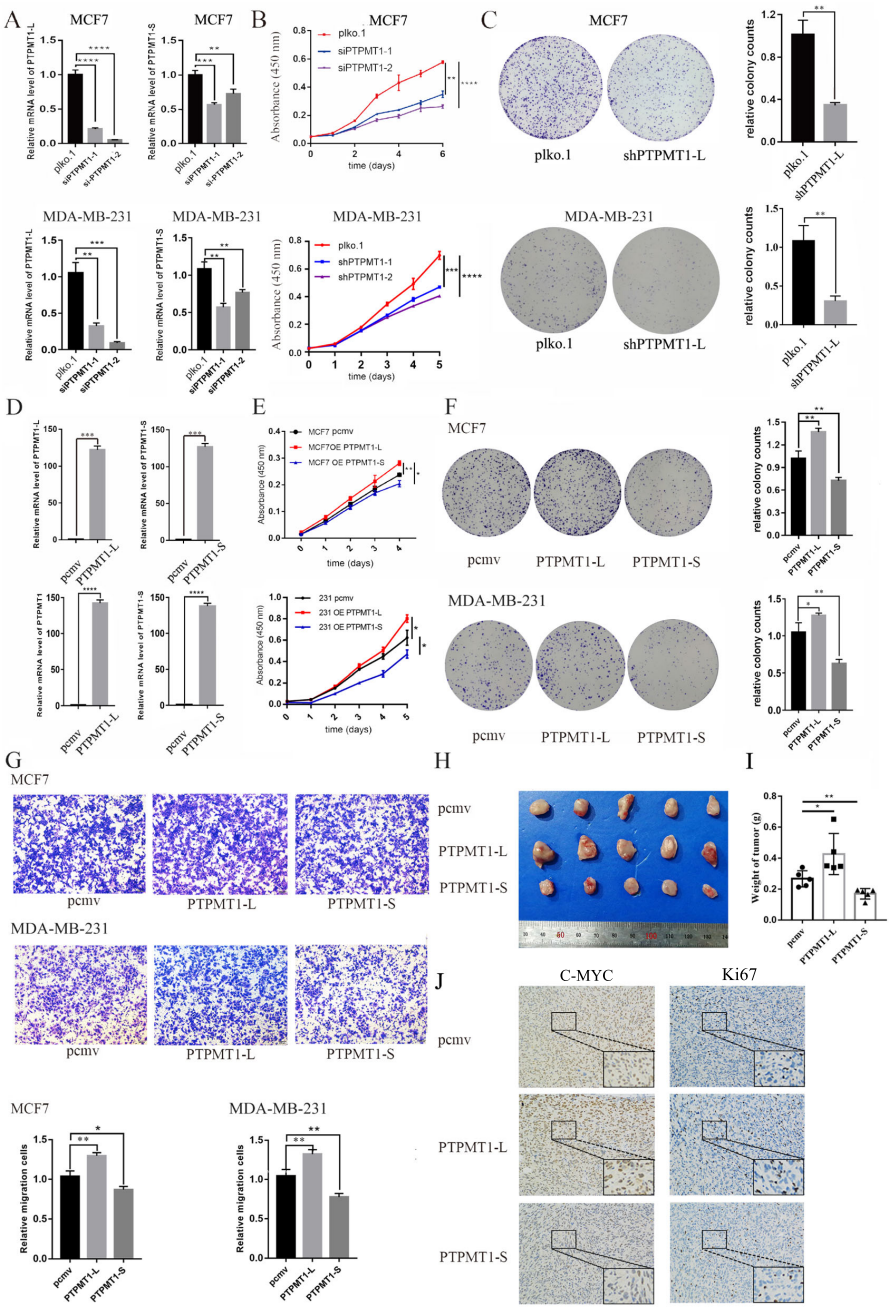
PTPMT1 splicing switch are required for cell growth and migration in vitro and in vivo. **a** qRT-PCR for testing the inhibitory effects of siPTPMT1-1/2 specific targeting to PTPMT1-L in MCF7/MDA-MB-231 cell lines. **b** Cell proliferation assay is performed using cells described in (**a**). **c** Clonogenic survival assay for control (pLKO.1) and treated (shPTPMT1-L) cells are conducted and then quantified in the bar graph. **d** qRT-PCR for testing overexpressing efficiency of PTPMT1-L/S plasmids in MCF7/MDA-MB-231 cell lines. **e**, **f** Cell proliferation assay and clonogenic survival assay for control (pCMV) and overexpression (PTPMT1-L/S) cells. **g** Measurement of cell migration by transwell assays using cells described in (**d**). **h** MDA-MB-231/PTPMT1-L/S cells or control MDA-MB-231/pCMV cells are transplanted to nude mice. Tumors derived from MDA-MB-231/PTPMT1-L/S or control cells are excised and presented. **i** Weight of tumors are calculated and plotted. **j** Representative IHC results of Ki67 and MYC in xenografts are presented (200*). Unpaired t-test. *, *P* < 0.05; ** *P* < 0.01; *** *P* < 0.001; **** *P* < 0.0001

To further investigate whether PTPMT1 splice switching affects the biological function of tumors, first, two siRNAs specifically targeting PTPMT1-L were constructed, and the inhibitory efficiency was confirmed by qRT-PCR (Fig. 6a, Supplementary Figure 3A). The results revealed that siPTPMT1-2 possessed higher specificity and efficiency in decreasing the expression of PTPMT1-L. Subsequent CCK-8 assays confirmed that both siRNAs inhibited MCF7/MDA-MB-231/T47D proliferation, while the inhibitory capacity of siPTPMT1-2 was more significant (Fig. 6b, Supplementary Figure 3B). Next, we constructed shPTPMT1-L plasmids by utilizing the sequence of siPTPMT1-2. Functional assays suggested that knockdown of PTPMT1-L isoforms significantly decreased the colony forming efficiency of MCF7/MDA-MB-231/T47D cells (Fig. 6c, Supplementary Figure 3C) and caused cell cycle arrest in G1 phase (Supplementary Figure 3D). Furthermore, PTPMT1-L/S plasmids were constructed and transfected into MCF7/T47D cells. The overexpression effect was confirmed by qRT-PCR (Fig. 6d, Supplementary Figure 3E). Intriguingly, CCK-8 (Fig. 6e, Supplementary Figure 3F) and colony formation assays (Fig. 6f, Supplementary Figure 3G) revealed that these two splice isoforms exerted opposite roles, as overexpression of PTPMT1-L promoted proliferation while overexpression of PTPMT1-S inhibited proliferation of MCF7/MDA-MB-231/T47D cells. In addition, overexpression of PTPMT1-L promoted migration, while overexpression of PTPMT1-S inhibited migration of MCF7/MDA-MB-231 cells (Fig. 6g). In vivo analysis confirmed that overexpression of PTPMT1-L significantly promoted tumorigenesis, while overexpression of PTPMT1-S inhibited tumor growth (Fig. 6h), as measured by the weight of tumors (Fig. 6i). Furthermore, IHC staining demonstrated that overexpression of PTPMT1-L increased the positive rate of the Ki-67 proliferation index, while overexpression of PTPMT1-S exerted the opposite effect (Fig. 6j). Collectively, these results indicate that PTPMT1-L promotes while PTPMT1-S inhibits the tumorigenic potential of BRCA cells in vivo and in vitro.

### SRSF1-regulated oncogenic activities partially mediated by aberrant PTPMT1 splice switching

**Fig. 7:**
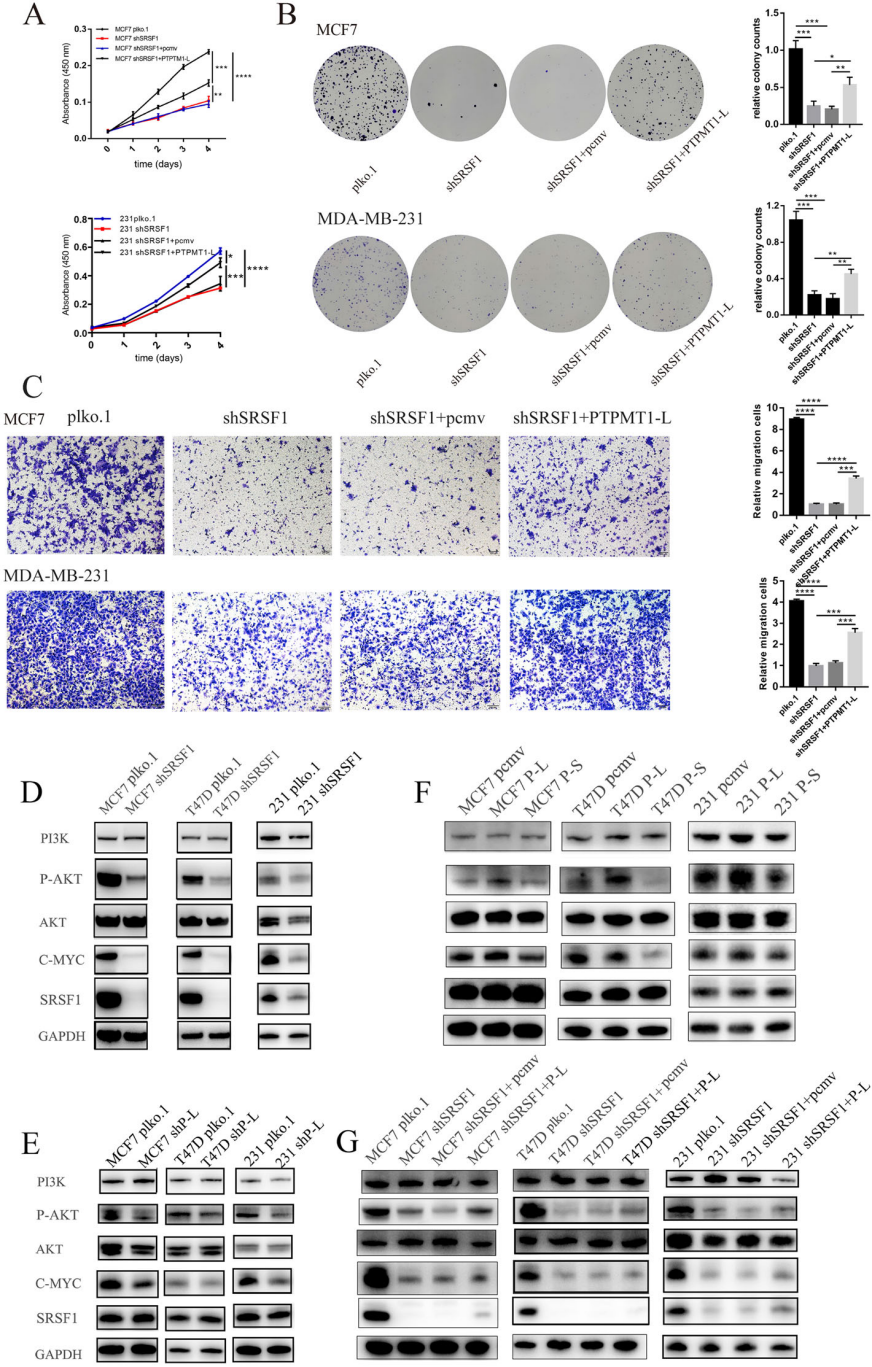
PTPMT1-L partially recapitulates the SRSF1-mediated tumor-promoting phenotypes through the P-AKT/C-MYC signaling pathways in breast cancer cells. **a**, **b** Cell proliferation assay (**a**) and clonogenic survival assay (**b**) of MCF7/MDA-MB-231 cells with depletion of SRSF1 or control, and re-expression of PTPMT1-L in SRSF1depleted cells are performed. **c** Measurement of cell migration by transwell assays using MCF7/MDA-MB-231 cells with depletion of SRSF1 or control, and re-expression of PTPMT1-L in SRSF1depleted cells. **d** Western blot for testing PI3K/AKT/C-MYC signaling pathways in MCF7/T47D/MDA-MB-231 cells with depletion of SRSF1 or control. **e** Western blot for testing PI3K/AKT/C-MYC signaling pathways in MCF7/T47D/MDA-MB-231 cells with depletion of PTPMT1-L or control. **f** Western blot for testing PI3K/AKT/C-MYC signaling pathways in MCF7/T47D/MDA-MB-231 cells with PTPMT1-L/S overexpression or control. **g** Western blot for testing PI3K/AKT/C-MYC signaling pathways in MCF7/T47D/MDA-MB-231 cells with depletion of SRSF1 or control, and re-expression of PTPMT1-L or control vector in SRSF1 depleted cells

Considering the biological function of SRSF1 in tumor cells, we investigated whether PTPMT1-L could partially mediate the oncogenic effects of SRSF1. We first established cells stably expressing shSRSF1, followed by infection with PTPMT1-L overexpression or control lentivirus. CCK-8 (Fig. 7a, Supplementary Figure 4A), colony formation (Fig. 7b, Supplementary Figure 4B) and Transwell assays (Fig. 7c) confirmed that the splicing isoform PTPMT1-L could partially rescue survival and migration defects caused by SRSF1 knockdown. Furthermore, we assessed the possible downstream mechanisms of the SRSF1-PTPMT1 splice switching axis. As mentioned above, KEGG enrichment analysis revealed that knockdown of SRSF1 significantly affected gene expression in the PI3K/AKT signaling pathways. Moreover, the PI3K/AKT signaling pathway plays oncogenic roles in many cancers, including breast cancer [40]. However, whether PTPMT1 splice switching exerts tumorigenic functions through the PI3K/AKT pathway remains unknown. Hence, we conducted western blotting assays and confirmed that knockdown of SRSF1 in MCF7/MDA-MB-231/T47D cells significantly decreased the protein expression levels of P-AKT and C-MYC (Fig. 7d). Knockdown of PTPMT1-L also decreased the protein expression levels of P-AKT and C-MYC (Fig. 7e). Interestingly, overexpression of PTPMT1-L increased P-AKT and C-MYC expression, while PTPMT1-S exerted the opposite effect (Fig. 7f). Moreover, PTPMT1-L overexpression followed by knockdown of SRSF1 partially rescued the inhibitory effect of SRSF1 on P-AKT and C-MYC (Fig. 7g). Collectively, our results demonstrate that SRSF1 might promote cancer cell growth partially through the SRSF1/PTPMT1 splice switching AKT/C-MYC signaling axis.

### Validation of SRSF1-mediated AS events in BRCA tissues and the TCGA dataset

**Fig. 8:**
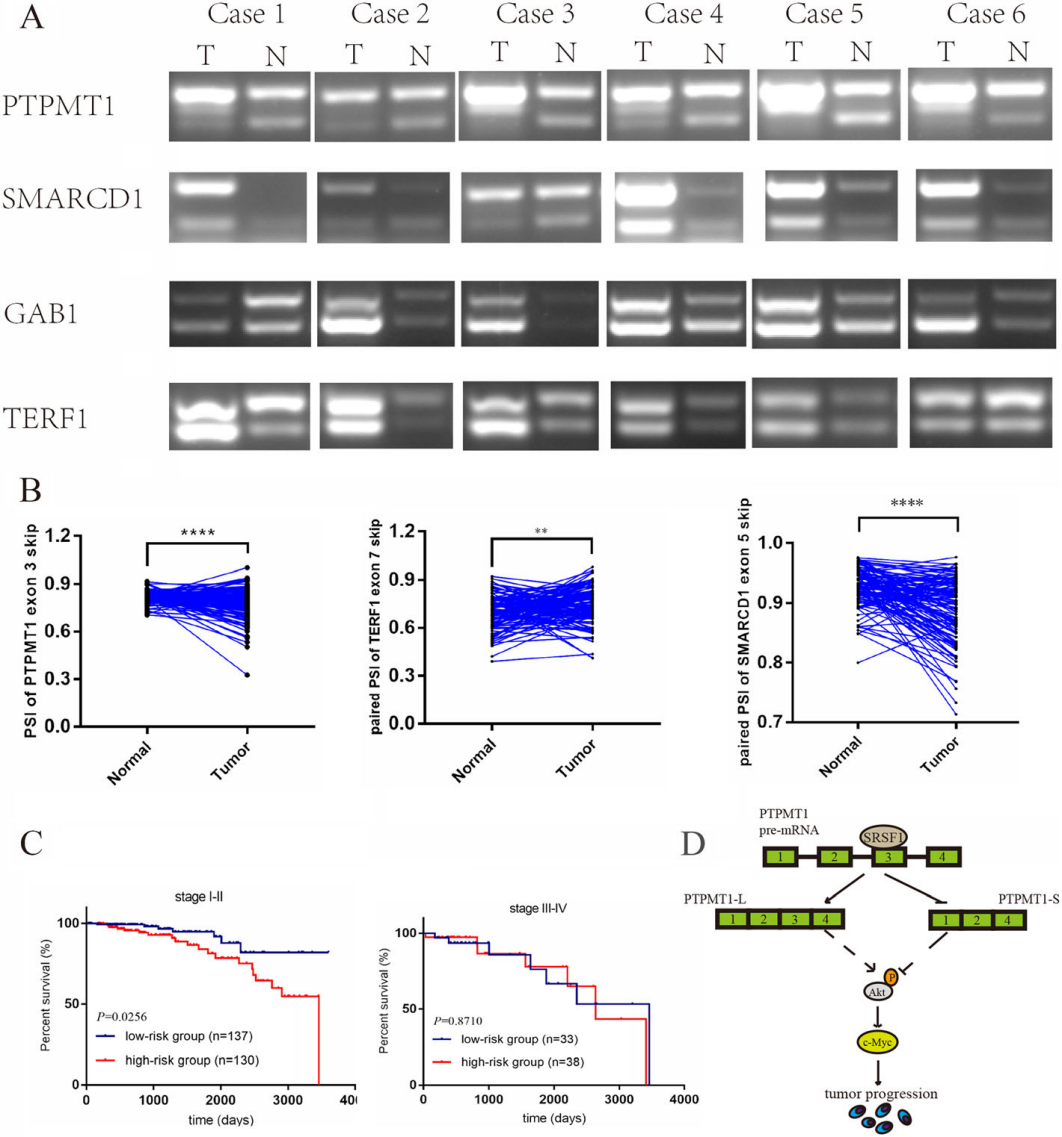
The expression patterns of representative splicing events regulated by SRSF1 in vivo and the clinical significance of splicing switching of PTPMT1. **a** Splicing pattern of PTPMT1/SMARCD1/GAB1/TERF1 in breast cancer and their paired adjacent tissues (*n* = 6) as detected by RT-PCR. **b** Comparison of the PSI of PTPMT1/TERF1/SMARCD1 in the 113 cancer and matched normal tissues from TCGA dataset. **c** Kaplan-Meier analysis of the OS of the high- (low PSI of PTPMT1 and high expression of SRSF1) and low-risk (high PSI of PTPMT1 and low expression of SRSF1) group in stage I-II or stage III-IV cohort based on TCGA data. The median of PSI of PTPMT1 or mRNA expression of SRSF1 are used as the cutoff. **d** The model of how SRSF1 mediates BRCA progression through modulating AS

To investigate SRSF1-mediated AS events in clinical BRCA samples, total RNA extracted from clinical BRCA tissues and RNA-seq data downloaded from the TCGA database were utilized for further analysis. Consistent with a previous analysis, a trend of an increased ratio of PTPMT1 E3 and SMARCD1 exon 5 (E5) inclusion isoforms was observed in tumor tissues compared to normal tissues (*n* = 6). Meanwhile, an increasing ratio of GAB1 exon 7 (E7) and TERF1 E7 exclusion isoforms was also observed (Fig. 8a). Because of the lower sequencing depths, the GAB1 E7 AS event was not included in the TCGA RNA-seq data. Consistent with our data, the ratio of PTPMT1 E3 and SMARCD1 E5 exclusion was decreased in tumor samples compared to paired normal samples, while TERF1 E7 exhibited the opposite AS trend (Fig. 8b). In addition, patients from the high-risk group identified by the expression of SRSF1 and PSI of PTPMT1 showed poorer prognosis than the low-risk group in patients with stage I-II disease (*P* = 0.0256), while there was no survival prediction significance in patients with stage III-IV disease (Fig. 8c). Collectively, SRSF1-mediated AS events identified by the high-throughput sequencing of cells also existed in BRCA tissue samples and possessed prognostic value in certain BRCA cohorts.

## Discussion

In this study, we conducted a comprehensive research on SRSF1 and its downstream profile of AS events in BRCA. We further confirmed that SRSF1 regulates pivotal splice switching of PTPMT1, which is correlated with BRCA tumorigenesis partially through the P-AKT/C-MYC axis (Fig. 8d). Collectively, our data confirmed that SRSF1 facilitates oncogenesis by regulating the AS of tumor-related genes, highlighting the significance of AS as a pivotal regulator of tumorigenesis.

SRSF1 as a classic member of the SR protein family, has been reported in several cancers and is associated with poor prognosis [22, 41]. Moreover, cardinal studies have shown that SRSF1 is upregulated in several tumors and exerts tumorigenesis roles by regulating the AS events of some cancer-related genes [42–44]. However, the clinical significance and biological function of SRSF1 in BRCA remain largely unknown. Hence, we confirmed that SRSF1 is upregulated in BRCA, and its upregulation correlates with higher BRCA malignancy features, such as proliferation index and histological grade and a poorer prognosis in HR+ patients. Further functional assays confirmed that knockdown of SRSF1 suppresses proliferation, cell migration, promotes cell cycle arrest and apoptosis in BRCA cells in vitro, and inhibits tumor growth in vivo. SRSF1 was overexpressed in human nontransformed mammary epithelial MCF10A cells in the previous study, and the phenotype of promoting cell proliferation and inhibiting apoptosis was observed [45], which was consistent with our research results. In addition, previous studies demonstrated that SRSF1 participated in the regulation of the cell cycle by preventing the formation of R loops between the new pre-mRNA and template DNA to maintain genome stability, which could support our results that knockdown SRSF1 in BRCA cell lines caused cell cycle arrest [46]. Moreover, recent studies have reported that the DCUN1D5 exon 4 inclusion regulated by SRSF1 promotes metastasis of BRCA cells [47]. Our study also showed that SRSF1 promoted cell migration of BRCA cells. Collectively, these findings suggest that SRSF1 is a pivotal driver of BRCA tumorigenesis, and indicate its prognostic value for HR+ BRCA patients.

RNA-seq analysis has identified numerous endogenous AS events regulated by SRSF1 [19, 21, 22], and constructed a sequence/position-dependent splicing signature [35, 42]. Nevertheless, the pivotal SRSF1-driven AS landscape responsible for oncogenesis usually differs largely in distinct tumor types [22, 42]. However, comprehensive studies focusing on SRSF1-controlled AS in BRCA have been limited until now. Here, by performing RNA-seq, we depicted SRSF1-controlled AS landscape in MCF7 cells. We used FDR < 0.05 and the PSI difference between the knockdown group and the control group ≥0.1 as the screening conditions. A total of 6699 AS events affected by the expression level of SRSF1 were found in MCF7 BRCA cell lines, of which SE was the most common splicing pattern, accounting for 75.2%. In the previous study, under the same screening conditions, using the model of MCF10A overexpressing SRSF1, 404 AS events regulated by SRSF1 were found, and SE splicing pattern accounted for 78.2%, which was partially consistent with our results [24]. Compared with previous studies, our sequencing results and analysis have found more AS events affected by SRSF1, and our splicing analysis covers 5 common splicing patterns, including MXE, which is not involved in previous studies [24]. It has been reported that the same SF plays a dual role in activating and repressing AS events [22, 24, 39]. Our sequencing results also portray this mode of regulation. It is worth noting that in the 5 AS patterns, the AS events repressed by SRSF1 accounted for more than 50% of the total. As a pivotal oncogene, SRSF1-regulated genes were enriched in autophagy, cell cycle checkpoint, cell cycle G2/M phase transition, mitochondrial organization biological functions and PI3K/AKT, p53, c-AMP and cancer-associated signaling pathways. Several significantly switching AS events identified by RNA-seq were then validated in MCF7/MDA-MB-231/T47D/293 T cells. Next, motif analysis, CLIP and minigene reporter assays were performed to investigate the regulatory mechanisms. Considering that SRSF1 promoted exon inclusion when binding motifs were enriched in cassette exons, and facilitated exon exclusion when binding motifs were enriched in the flanking constitutive exons and introns, we discovered that SRSF1 regulated exon inclusion/exclusion in a position-dependent manner. Moreover, there are currently three main methods for predicting binding sites, including motifs derived from data from SELEX [48] or CLIP experiments [35] and motifs derived from RNA-seq [22, 24]. Previous analysis showed that the predictive power of RNA-seq-derived motifs is comparable to the former two [24]. Then two deletion mutant plasmids in our study were constructed based on previously reported SELEX and CLIP data (AGGAG/GAAGA) [35, 48] and the predicted SRSF1 binding motif derived from RNA-seq (part of which coincides with the previously reported motif sequence, as shown in yellow in Fig. 5a), respectively. The results showed that in the AS regulation of PTPMT1 by SRSF1, the former was a more effective binding site. In reports on radiotherapy for lung cancer, the binding site regulated by SRSF1 was TTACCAGTAA [21], and another report predicted and verified that the binding motif of SRSF1 derived from RNA-seq to regulate the AS of MYO1B is GAGGGG [49]. The common feature of these sequences, including the motifs predicted by our RNA-seq, is that they are GA-rich. In addition, studies have shown that when SRSF1 promotes exon inclusion, its binding motif tends to be in the cassette exon near the 5′SS [24]. The result of our minigene reporter assay is consistent with it, suggesting that when SRSF1 regulates the AS of PTPMT1, its binding site is sequence- and position-dependent. Besides, among these AS events regulated by SRSF1 in BRCA, we found that PTPMT1, SMARCD1, GAB1 and TERF1 were directly bound and regulated by SRSF1. Specifically, SRSF1 facilitated the inclusion of PTPMT1 E3/ SMARCD1 E5, and promoted the exclusion of GAB1 E7/ TERF1 E7.

PTPMT1 is a dual specificity phosphatase that predominantly localizes to mitochondria and the nucleus. PTPMT1 pre-mRNA is spliced into two splicing isoforms, PTPMT1-L and PTPMT1-S. PTPMT1-L represents the long transcript containing 4 exons, and its encoded protein contains the PTP (protein tyrosine phosphatases) conserved domain. The PTP domain catalyzes the dephosphorylation of phosphotyrosine peptides to regulate the level of phosphotyrosine in the signal transduction pathway. The short transcript, PTPMT1-S, contains 3 exons. The lack of exon 3 results in a frameshift, generating a protein with a different C-terminus and no PTP conserved domain. Previous studies found that PTPMT1 inhibited apoptosis and promoted proliferation in cancer cells, indicating the oncogenic role of PTPMT1 [50, 51]. In addition, PTPMT1 is pivotal for the differentiation of hematopoietic stem cells (HSCs) by activating AMPK [52]. More intriguingly, it has been confirmed that PTPMT1-S promotes phosphorylation of AMPK, thereby promoting irradiation sensitivity in lung cancer [21]. In agreement with these studies, we found that PTPMT1-L promoted BRCA progression by facilitating proliferation, cell migration, resulting in cell cycle arrest of MCF7/MDA-MB-231/T47D cells, while PTPMT1-S exerted opposite functions. Moreover, PTPMT1-L partially rescued the oncogenic functions of SRSF1-silenced BRCA cells. Since we validated the PTPMT1 AS events in clinical samples and TCGA datasets, we cautiously conclude that SRSF1 mediates oncogenesis and progression of BRCA partially by regulating the splice switching of PTPMT1.

Then, we explored the downstream pathways mediating the oncogenic function of SRSF1 and AS of PTPMT1. KEGG enrichment analysis indicated that the PI3K/AKT signaling pathway was regulated by SRSF1. Importantly, the PI3K/AKT pathway plays pivotal roles in BRCA progression, drug resistance and treatment. However, whether the AS of PTPMT1 affects this pathway and the regulatory relationship of the SRSF1/AS of the PTPMT1/PI3K/AKT signaling pathway remain unknown. Our data confirmed that knockdown of SRSF1 severely inhibits the phosphorylation of AKT and the expression of C-MYC. Intriguingly, overexpression of PTPMT1-L facilitates the phosphorylation of AKT and expression of C-MYC, while PTPMT1-S exerts opposite regulatory functions. Moreover, overexpression of PTPMT1-L partially rescues the repressed effect caused by SRSF1 silencing. These data indicated that SRSF1/AS of the PTPMT1/P-AKT/C-MYC pathway regulatory axis exists in BRCA cells. Based on a previous study suggesting that C-MYC, as a transcription factor promotes the transcription of SRSF1 [53], we cautiously speculate that positive regulatory feedback exists between SRSF1 and C-MYC.

## Conclusion

Taken together, our data herein confirm that SRSF1 serves as an oncogene in BRCA by promoting proliferation, cell migration and inhibiting cell cycle arrest and apoptosis. Moreover, SRSF1 was positively correlated with malignant BRCA features, including the Ki-67 index and histological tumor grade in BRCA and poor prognosis in HR+ BRCA patients. SRSF1 regulates AS events in a position-dependent manner and promotes the generation of PTPMT1-L by binding to the motifs in E3. The splice switching of PTPMT1 regulated by SRSF1 serves as an oncogenic factor for BRCA. In addition, an SRSF1/PTPMT1/P-AKT/C-MYC pathway regulatory axis exists in BRCA cells. SRSF1 and PTPMT1-L have the potential to be novel prognostic biomarkers and therapeutic targets for BRCA therapy.

## Supplementary Information

**Additional file 1: Supplementary Figure 1.** Knockdown of SRSF1 suppresses oncogenic roles in T47D cells. (A) Luminal subtype cell line T47D is transfected with SRSF1 shRNA plasmid (shSRSF1) or control plasmid (pLKO.1). SRSF1 knockdown efficiency is confirmed by western blot and RT-qPCR. (B, C) Cell proliferation assay and clonogenic survival assay are performed using cells described in (A). (D) The cell cycle of cells described in (A) is analyzed by flow cytometry and the relative cell population of each cell cycle phase is quantified in the bar graph. (E) The ration of apoptosis in each group is calculated by flow cytometry.

**Additional file 2: Supplementary Figure 2.** Validation of representative SRSF1-affected SE events in T47D and 293Tcells. (A) qRT-PCR and western blot for testing the overexpression efficiency of SRSF1-OE plasmids in 293 T cells. (B) Representative SRSF1-affected SE events tested in T47D and 293 T cells, RT-PCR results and quantification of their RNA products measured as inclusion/exclusion (In/Ex).

**Additional file 3: Supplementary Figure 3.** PTPMT1 splicing switch are required for cell growth and migration in vitro. (A) qRT-PCR for testing the inhibitory effects of siPTPMT1-1/2 specific targeting to PTPMT1-L in T47D cell lines. (B) Cell proliferation assay is performed using cells described in (A). (C) Clonogenic survival assay for control (pLKO.1) and treated (shPTPMT1-L) cells are conducted and then quantified in the bar graph. (D) The cell cycle of control (pLKO.1) and treated (shPTPMT1-L) cells are analyzed by flow cytometry and the relative cell population of each cell cycle phase is quantified in the bar graph. (E) qRT-PCR for testing overexpressing efficiency of PTPMT1-L/S plasmids in T47D cell line. (F, G) Cell proliferation assay and clonogenic survival assay for control (pCMV) and overexpression (PTPMT1-L/S) cells. (H) Measurement of cell migration by wound-healing assays using MCF7/MDA-MB-231 cells transfected with PTPMT1-L/S or control plasmids.

**Additional file 4: Supplementary Figure 4.** PTPMT1-L partially recapitulates the SRSF1-mediated tumor-promoting phenotypes inT47D cells. (A, B) Cell proliferation assay (A) and clonogenic survival assay (B) of T47D cells with depletion of SRSF1 or control, and re-expression of PTPMT1-L in SRSF1depleted cells are performed.

**Additional file 5: Supplementary Table 1**

**Additional file 6: Supplementary Table 2**

**Additional file 7: Supplementary Table 3**

**Additional file 8: Supplementary Table 4**

**Additional file 9: Supplementary Table 5**

**Additional file 10: Supplementary Table 6**

**Additional file 11: Supplementary Materials**

## Data Availability

All data generated or analyzed during this study are included in this published article and its supplementary information files.

## Abbreviations

AS: Alternative splicing
BRCA: Breast cancer
RIP-PCR: RNA immunoprecipitation-PCR
CLIP: Crosslinking followed by immunoprecipitation
E3: Exon 3
HR +: Hormone receptor positive
SE: Skipped exon
IR: Intron retention
MXE: Mutually exclusive exon
A5SS/A3SS: Alternative 5′ or 3′ splice site
UTRs: Untranslated regions
AFE/ALE: Alternative first or last exon
snRNA: Small nuclear ribonucleic acids
snRNP: Small nuclear ribonucleoprotein
SF: Splicing factor
SRE: Splicing regulatory element
RRM: RNA recognition motif
RS: Arg/Ser-rich
IHC: Immunohistochemistry
OS: Overall survival
DEGs: Differentially expressed genes
E5: Exon 5
E7: Exon7
PTP: Protein tyrosine phosphatases
HSCs: Hematopoietic stem cells
