# Supplementary figures and images for: Splicing factor SRSF1 promotes breast cancer progression via oncogenic splice switching of PTPMT1
Source: J Exp Clin Cancer Res. 2021 May 15;40:171. doi: 10.1186/s13046-021-01978-8 (PMC8122567; doi:10.1186/s13046-021-01978-8)

A

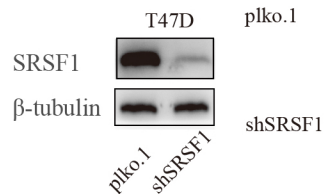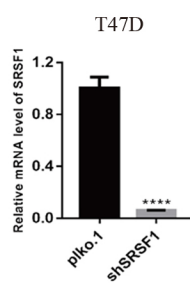

B

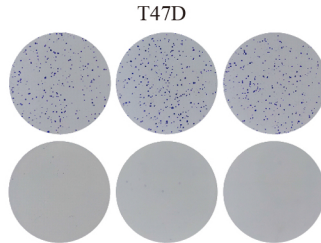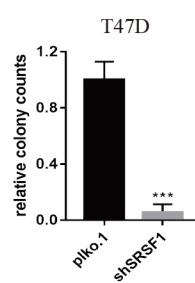

C

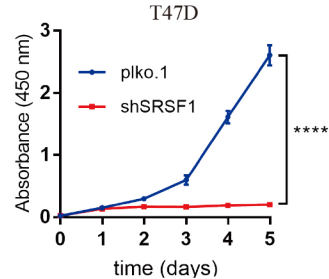

D

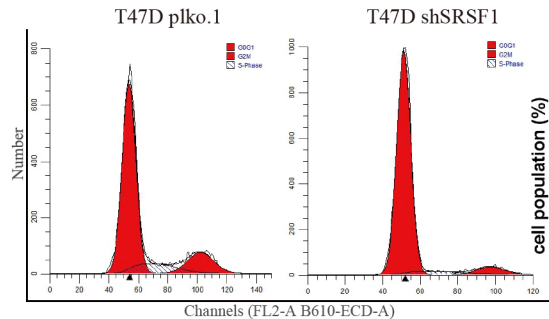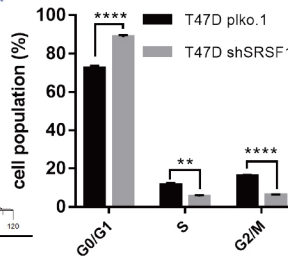

E

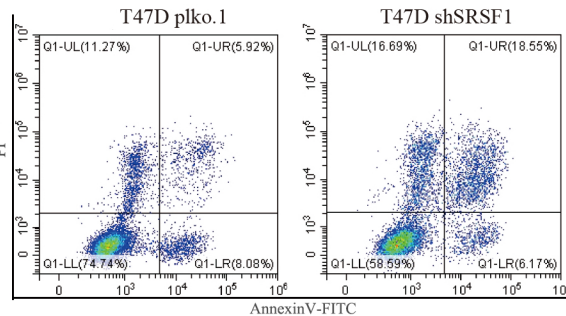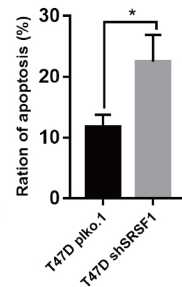

Supplement: Supplementary file 1 — Additional file 1: Supplementary Figure 1. Knockdown of SRSF1 suppresses oncogenic roles in T47D cells. (A) Luminal subtype cell line T47D is transfected with SRSF1 shRNA plasmid (shSRSF1) or control plasmid (pLKO.1). SRSF1 knockdown efficiency is confirmed by western blot and RT-qPCR. (B, C) Cell proliferation assay and clonogenic survival assay are performed using cells described in (A). (D) The cell cycle of cells described in (A) is analyzed by flow cytometry and the relative cell population of each cell cycle phase is quantified in the bar graph. (E) The ration of apoptosis in each group is calculated by flow cytometry. [file 13046_2021_1978_MOESM1_ESM.pdf]

A

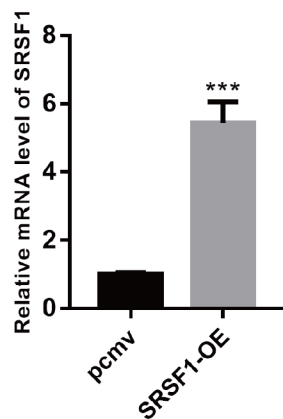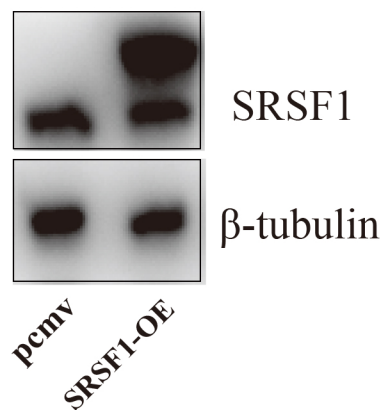

B

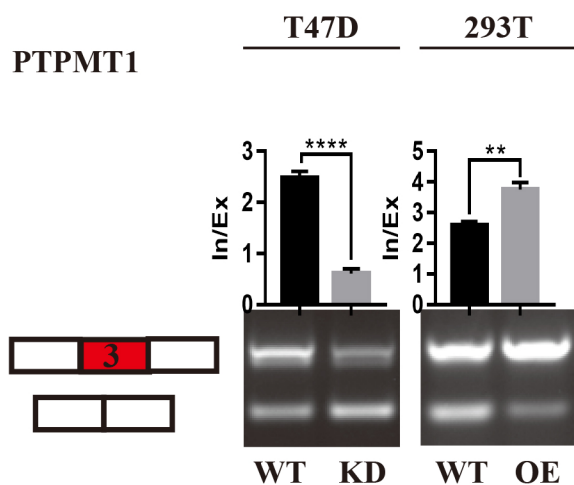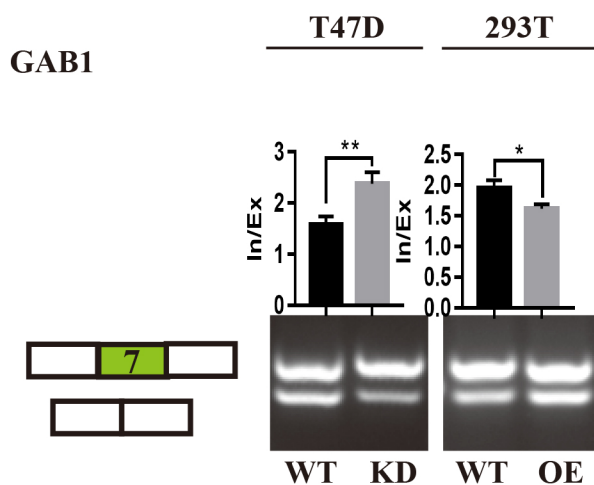

SMARCD1

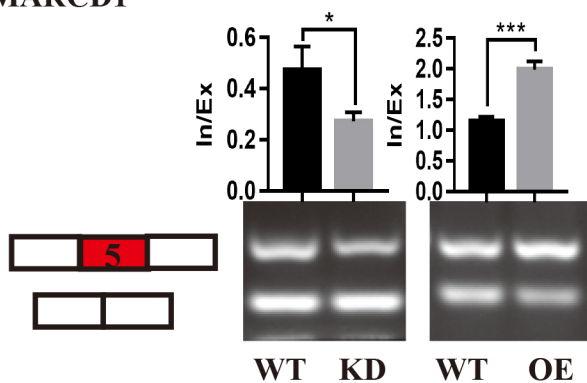

TERF1

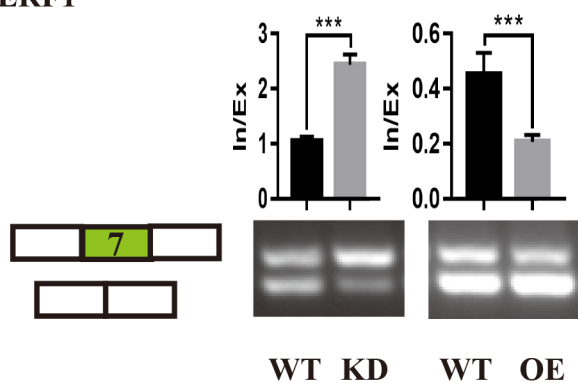

Supplement: Supplementary file 2 — Additional file 2: Supplementary Figure 2. Validation of representative SRSF1-affected SE events in T47D and 293Tcells. (A) qRT-PCR and western blot for testing the overexpression efficiency of SRSF1-OE plasmids in 293 T cells. (B) Representative SRSF1-affected SE events tested in T47D and 293 T cells, RT-PCR results and quantification of their RNA products measured as inclusion/exclusion (In/Ex). [file 13046_2021_1978_MOESM2_ESM.pdf]

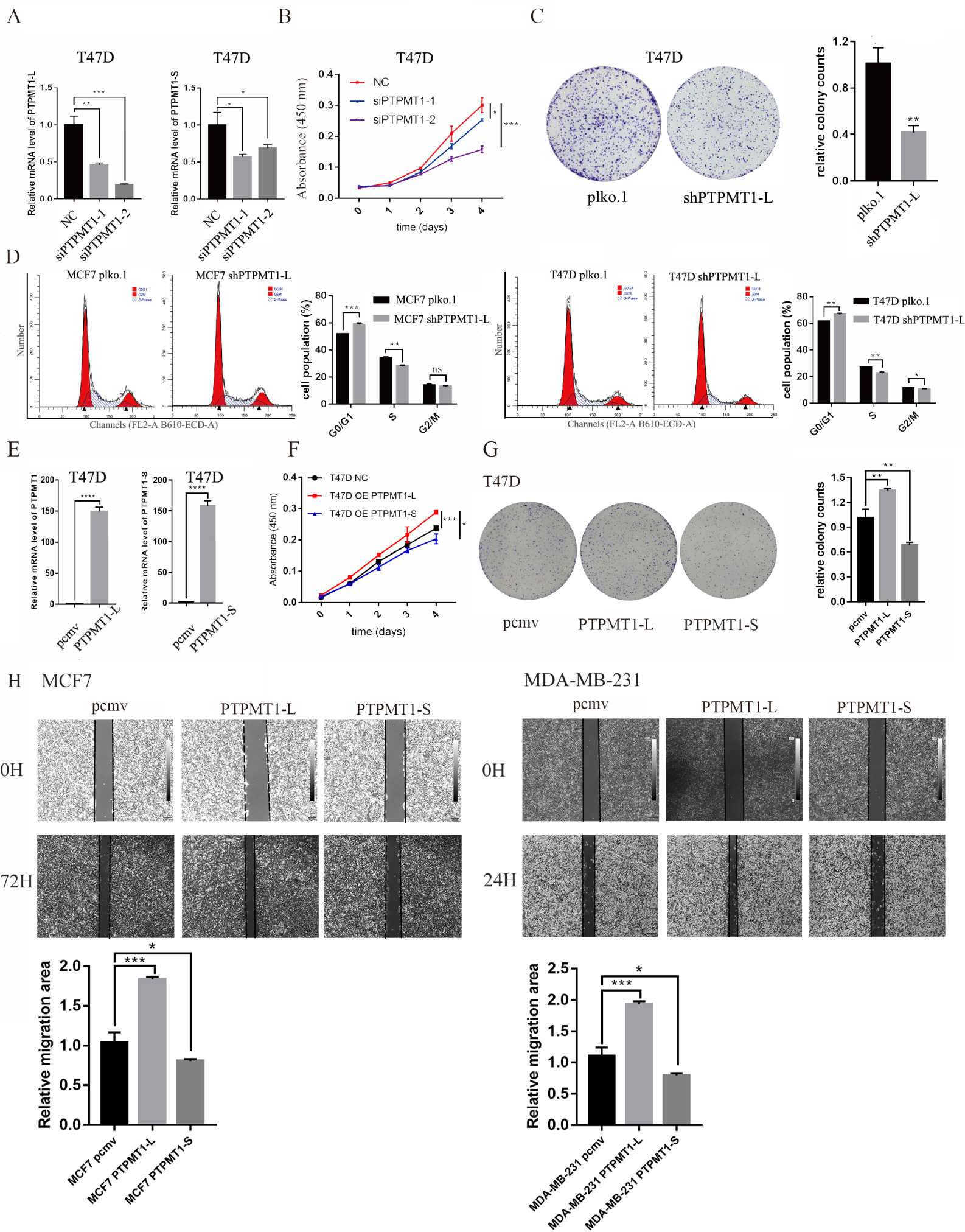

Supplement: Supplementary file 3 — Additional file 3: Supplementary Figure 3. PTPMT1 splicing switch are required for cell growth and migration in vitro. (A) qRT-PCR for testing the inhibitory effects of siPTPMT1-1/2 specific targeting to PTPMT1-L in T47D cell lines. (B) Cell proliferation assay is performed using cells described in (A). (C) Clonogenic survival assay for control (pLKO.1) and treated (shPTPMT1-L) cells are conducted and then quantified in the bar graph. (D) The cell cycle of control (pLKO.1) and treated (shPTPMT1-L) cells are analyzed by flow cytometry and the relative cell population of each cell cycle phase is quantified in the bar graph. (E) qRT-PCR for testing overexpressing efficiency of PTPMT1-L/S plasmids in T47D cell line. (F, G) Cell proliferation assay and clonogenic survival assay for control (pCMV) and overexpression (PTPMT1-L/S) cells. (H) Measurement of cell migration by wound-healing assays using MCF7/MDA-MB-231 cells transfected with PTPMT1-L/S or control plasmids. [file 13046_2021_1978_MOESM3_ESM.pdf]

A

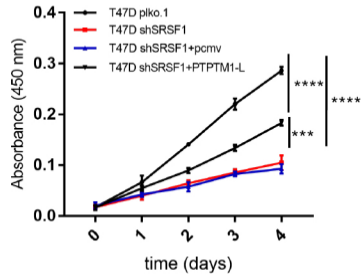

B

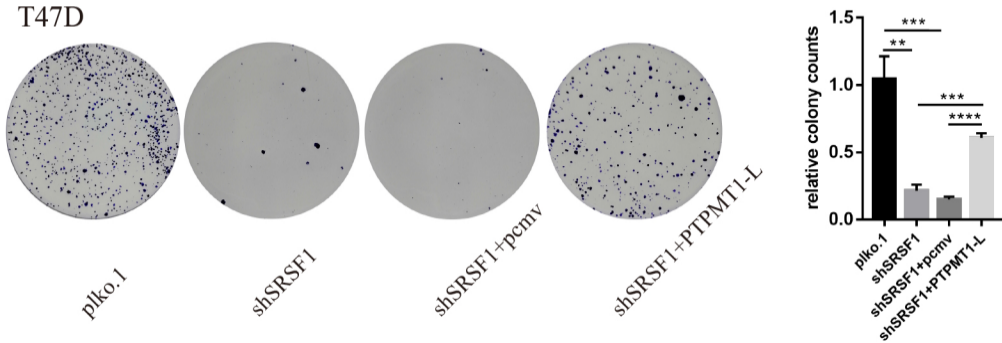

Supplement: Supplementary file 4 — Additional file 4: Supplementary Figure 4. PTPMT1-L partially recapitulates the SRSF1-mediated tumor-promoting phenotypes inT47D cells. (A, B) Cell proliferation assay (A) and clonogenic survival assay (B) of T47D cells with depletion of SRSF1 or control, and re-expression of PTPMT1-L in SRSF1depleted cells are performed. [file 13046_2021_1978_MOESM4_ESM.pdf]
