# Supplementary material for: Splicing factor SRSF1 promotes breast cancer progression via oncogenic splice switching of PTPMT1
Source: J Exp Clin Cancer Res. 2021 May 15;40:171. doi: 10.1186/s13046-021-01978-8 (PMC8122567; doi:10.1186/s13046-021-01978-8)
Supplement: Supplementary file 5 — Additional file 5: Supplementary Table 1 [file 13046_2021_1978_MOESM5_ESM.docx]

**Supplementary table 1. Primary antibodies for Western blot of IHC staining.**

| Name | Catlog No. | Company |
| --- | --- | --- |
| mouse anti-human SRSF1 | sc-33652 | Santa Cruz Biotechnology |
| mouse anti-human β-tubulin | M1305-2 | Hangzhou HuaAn Biotechnology Co.,Ltd |
| mouse anti-human GAPDH | ab8245 | Abcam |
| rabbit anti-human p85 PI3K | A4992 | Abclonal |
| rabbit anti-human AKT1 | A17909 | ABclonal |
| rabbit anti-human S473 AKT1 | AP0637 | ABclonal |
| rabbit anti-human c-Myc | A19032 | ABclonal |
| mouse anti-human KI-67 | sc-23900 | Santa Cruz Biotechnology |
