# Supplementary material for: Splicing factor SRSF1 promotes breast cancer progression via oncogenic splice switching of PTPMT1
Source: J Exp Clin Cancer Res. 2021 May 15;40:171. doi: 10.1186/s13046-021-01978-8 (PMC8122567; doi:10.1186/s13046-021-01978-8)
Supplement: Supplementary file 6 — Additional file 6: Supplementary Table 2 [file 13046_2021_1978_MOESM6_ESM.docx]

**Supplementary table 2. Sequences of siRNAS and shRNAs**

| Name | Sequences |
| --- | --- |
| si-NC-Sense | 5’-GGUGGAACAAUUGCUUUUAdTdT-3’ |
| si-NC-Antisense | 5’-UAAAAGCAAUUGUUCCACCdTdT-3’ |
| si-PTPMT1-L-1#-Sense | 5'-GGCUCAGCACAGUAGACAUGATT-3' |
| si-PTPMT1-L-1#-Antisense | 5'-UCAUGUCUACUGUGCUGAGCCTT-3' |
| si-PTPMT1-L-2#-Sense | 5'-GGUGGCAGCAUACCUGAUUCATT-3' |
| si-PTPMT1-L-2#-Antisense | 5'-UGAAUCAGGUAUGCUGCCACCTT-3' |
| sh-NC-Sense | 5’-ccggTTCCTGGAACAATTGCTTTTACTCGAGTAAAAGCAATTGTTCCAGGAATTTTTg-3’ |
| sh-NC-Antisense | 5’-aattcAAAAATTCCTGGAACAATTGCTTTTACTCGAGTAAAAGCAATTGTTCCAGGAA-3’ |
| sh-SRSF1-Sense | 5'-ccggGCTGATGTTTACCGAGATGGCCTCGAGGCCATCTCGGTAAACATCAGCtttttga-3' |
| sh-SRSF1-Antisense | 5'-aattcaaaaaGCTGATGTTTACCGAGATGGCCTCGAGGCCATCTCGGTAAACATCAGC-3' |
| sh-PTPMT1-L-Sense | 5’-ccggGGTGGCAGCATACCTGATTCACTCGAGTGAATCAGGTATGCTGCCACCtttttga-3’ |
| sh-PTPMT1-L-Antisense | 5’-aattcaaaaaGGTGGCAGCATACCTGATTCACTCGAGTGAATCAGGTATGCTGCCACC-3’ |
