# Supplementary material for: Splicing factor SRSF1 promotes breast cancer progression via oncogenic splice switching of PTPMT1
Source: J Exp Clin Cancer Res. 2021 May 15;40:171. doi: 10.1186/s13046-021-01978-8 (PMC8122567; doi:10.1186/s13046-021-01978-8)
Supplement: Supplementary file 7 — Additional file 7: Supplementary Table 3 [file 13046_2021_1978_MOESM7_ESM.docx]

**Supplementary table 3. Sequences of qRT-PCR primers**

| Name | Sequences |
| --- | --- |
| SRSF1-Forward | 5’- GCCGCATCTACGTGGGTAAC-3’ |
| SRSF1-Reverse | 5’- GAGGTCGATGTCGCGGATAG-3’ |
| ACTB-Forward | 5’-GATCATTGCTCCTCCTGAGC-3’ |
| ACTB-Reverse | 5’-ACTCCTGCTTGCTGATCCAC-3’ |
| 18S-Forward | 5'-CGGCGACGACCCATTCGAAC-3' |
| 18s-Reverse | 5'-GAATCGAACCCTGATTCCCCGTC-3' |
| PTPMT1-L-Forward | 5‘-GCTCTCAAGTACCAGTCGCT-3’ |
| PTPMT1-L-Reverse | 5‘-ATGACCGGATCTTGGCGATG-3’ |
| PTPMT1-S-Forward | 5‘-GTCTCCACCGTCTTTGCTGA-3’ |
| PTPMT1-S-Reverse | 5‘-TTGCACAGGAACCTCGTCTC-3’ |
