# Supplementary material for: Splicing factor SRSF1 promotes breast cancer progression via oncogenic splice switching of PTPMT1
Source: J Exp Clin Cancer Res. 2021 May 15;40:171. doi: 10.1186/s13046-021-01978-8 (PMC8122567; doi:10.1186/s13046-021-01978-8)
Supplement: Supplementary file 8 — Additional file 8: Supplementary Table 4 [file 13046_2021_1978_MOESM8_ESM.docx]

Supplementary table 4. Sequences of RT-PCR primers

| Primers for AS events |  |
| --- | --- |
| Name | Sequences |
| PTPMT1-Forward | 5'-TGGTACAGGACGAGAACGTG-3' |
| PTPMT1-Reverse | 5'-GGATCTTGGCGATGGCTCTT-3' |
| FER-Forward | 5'-CCAAAATCTGCACTGGGCTC-3' |
| FER-Reverse | 5'-GAAAACCCAGTGCCCTCGAA-3' |
| SMARCD1-Forward | 5'-GAGGCCTTGAAACGTCCCAT-3' |
| SMARCD1-Reverse | 5'-GCCCATACAGGTCTTTGTCCA-3' |
| NAV1-Forward | 5'-GTACCAGCTTCAGTCCCAGG-3' |
| NAV1-Reverse | 5'-AGTAGGTGGAGGAGGCACT-3' |
| GAB1-Forward | 5'-ACCACGACAACATTCCAGCA-3' |
| GAB1-Reverse | 5'-GGGAGATCTAACTGGGGCTTG-3' |
| HDAC7-Forward | 5'-CCCCAAGTAGTAGCAGCACG-3' |
| HDAC7-Reverse | 5'-GAGGGGTCCAGGAGGAGAAT-3' |
| TERF1-Forward | 5'-GCGGCAAAAGTAGTAGAAAGCA-3' |
| TERF1-Reverse | 5'-GGTCTTGTTGCTGGGTTCCA-3' |
| MAPK11-Forward | 5'-CCCTTCCAGTCGCTGATCCA-3' |
| MAPK11-Reverse | 5'-TGACGATGTTGTTCAGGTCGG-3' |
| PTPMT1-E2-Forward | 5'-GTCTCCACCGTCTTTGCTGA-3' |
| PTPMT1-E2-Reverse | 5'-CTGTGAAGAGTTGCACAGGAAC-3' |
| PTPMT1-E3-Forward | 5'-GCGGCTCAGCACAGTAGACAT-3' |
| PTPMT1-E3-Reverse | 5'-GCCTTACAATGCACGTAAACA-3' |
| PTPMT1-E4-Forward | 5'-GTAAGAGCCATCGCCAAGA-3' |
| PTPMT1-E4-Reverse | 5'-AATGACAAAAGTCCCATCC-3' |
| SMARCD1-E4-Forward | 5'-GTACCAGAATCCCAGGCCTAT-3' |
| SMARCD1-E4-Reverse | 5'-TGATGGGACGTTTCAAGGC-3' |
| SMARCD1-E5-Forward | 5'-CTGCGAATTTTCATTTCTAAC-3' |
| SMARCD1-E5-Reverse | 5'-AGGAGCCGTCCTTCTACCC-3' |
| SMARCD1-E6-Forward | 5'-TCAGCCTTGTCCAAATATG-3' |
| SMARCD1-E6-Reverse | 5'-TTGTCCAGTTCAATCACCA-3' |
| GAB1-E6-Forward | 5'-GTGTTGACAGTGGGAAGTG-3" |
| GAB1-E6-Reverse | 5'-CTGTCTGGCTTGAGGTTC-3' |
| GAB1-E7-Forward | 5'-TTTTAAGACTCAAACCCC-3' |
| GAB1-E7-Reverse | 5'-CCTTTCTTCTTGTAGCAA-3' |
| GAB1-E8-Forward | 5'-GCCTTTAGAAATAAAACCT-3' |
| GAB1-E8-Reverse | 5'-CGAGCAAAACTCCTAGTGA-3' |
| TERF1-E6-Forward | 5'-AGCGGCAAAAGTAGTAGAA-3' |
| TERF1-E6-Reverse | 5'-TATCCAAATTAGCTTCAGT-3' |
| TERF1-E7-Forward | 5'-AAATTGAAATATGCCTTGAC-3' |
| TERF1-E7-Reverse | 5'-TACCCTCTGAGGATTCAGTTAC-3' |
| TERF1-E8-Forward | 5'-GTCTCACAAGAATCTTTTC-3' |
| TERF1-E8-Reverse | 5'-AGGAGTTCCTACTCTTCTT-3' |
| PTPMT1-minigene-Forward | 5‘-TGGCTAGCCTGGTACAGGACGAGAA-3’ |
| PTPMT1-minigene-Reverse | 5‘-CGGATCTTGGCGATGGCTCT-3’ |
| Primers for RIP-PCR |  |
| Name | Sequences |
| PTPMT1-Forward | 5'-GCTCTCAAGTACCAGTCGCT-3' |
| PTPMT1-Reverse | 5'-ATGACCGGATCTTGGCGATG-3' |
| SMARCD1-Forward | 5'-TCAGTCCCGCAAGAGACCT-3' |
| SMARCD1-Reverse | 5'-CCAGTTCACGAATCCTTTGAGG-3' |
| GAB1-Forward | 5'-TGGCAGCTCTTTACAAGCACC-3' |
| GAB1-Reverse | 5'-TGATGAGCAACAGGTAGTCTTGA-3' |
| TERF1-Forward | 5'-AACAGCGCAGAGGCTATTATTC-3' |
| TERF1-Reverse | 5'-CCAAGGGTGTAATTCGTTCATCA-3' |
| GAPDH-Forward | 5'-GGAGCGAGATCCCTCCAAAAT-3' |
| GAPDH-Reverse | 5'-GGCTGTTGTCATACTTCTCATGG-3' |
