# Supplementary material for: Splicing factor SRSF1 promotes breast cancer progression via oncogenic splice switching of PTPMT1
Source: J Exp Clin Cancer Res. 2021 May 15;40:171. doi: 10.1186/s13046-021-01978-8 (PMC8122567; doi:10.1186/s13046-021-01978-8)
Supplement: Supplementary file 9 — Additional file 9: Supplementary Table 5 [file 13046_2021_1978_MOESM9_ESM.docx]

Supplementary table 5. Sequences of deletion mutation

| Name | Sequences |
| --- | --- |
| E3F1-F | CTGGTTCCAGGAGTGTCGAGCAGCTGCGGC |
| E3F1-R | GCCGCAGCTGCTCGACACTCCTGGAACCAG |
| E3F2-F | CAATTTGCTCTCAAGTATGCCACTATGGTGGC |
| E3F2-R | GCCACCATAGTGGCATACTTGAGAGCAAATTG |
