# Supplementary material for: Splicing factor SRSF1 promotes breast cancer progression via oncogenic splice switching of PTPMT1
Source: J Exp Clin Cancer Res. 2021 May 15;40:171. doi: 10.1186/s13046-021-01978-8 (PMC8122567; doi:10.1186/s13046-021-01978-8)
Supplement: Supplementary file 10 — Additional file 10: Supplementary Table 6 [file 13046_2021_1978_MOESM10_ESM.docx]

|  | **chr** | **exonStart_0base** | **exonEnd** | **upstreamES** | **upstreamEE** | **downstreamES** | **downstreamEE** | **PValue** | **FDR** | **PSI difference** |
| --- | --- | --- | --- | --- | --- | --- | --- | --- | --- | --- |
| PTPMT1 | 11 | 47569699 | 47569891 | 47565457 | 47565986 | 47571470 | 47571769 | 0 | 0 | 0.304 |
| SMARCD1 | 12 | 50087362 | 50087485 | 50086755 | 50086878 | 50088520 | 50088637 | 7.64E-10 | 4.45E-08 | 0.31 |
| FER | 5 | 108954728 | 108954932 | 108946129 | 108946222 | 108959224 | 108959347 | 0.00129 | 0.01431964 | 0.705 |
| NAV1 | 1 | 201788467 | 201788638 | 201786428 | 201786577 | 201789739 | 201789792 | 8.66E-07 | 2.62E-05 | 0.598 |
| GAB1 | 4 | 143457679 | 143457769 | 143440078 | 143440382 | 143459384 | 143459429 | 0.000604 | 0.00757998 | -0.38 |
| HDAC7 | 12 | 47795905 | 47796016 | 47795586 | 47795767 | 47796206 | 47796298 | 2.41E-06 | 6.48E-05 | -0.366 |
| TERF1 | 8 | 73030335 | 73030395 | 73026939 | 73027052 | 73032041 | 73032133 | 0 | 0 | -0.485 |
| MAPK11 | 22 | 50267568 | 50267627 | 50267370 | 50267482 | 50267819 | 50267949 | 5.57E-07 | 1.75E-05 | -0.488 |

Supplementary table 6. Detail information of representative AS events.
