# Supplementary material for: Splicing factor SRSF1 promotes breast cancer progression via oncogenic splice switching of PTPMT1
Source: J Exp Clin Cancer Res. 2021 May 15;40:171. doi: 10.1186/s13046-021-01978-8 (PMC8122567; doi:10.1186/s13046-021-01978-8)
Supplement: Supplementary file 11 — Additional file 11: Supplementary Materials [file 13046_2021_1978_MOESM11_ESM.docx]

**Supplemental materials**

**Immunohistochemistry (IHC)**

For IHC assay, tissue sections were deparaffinized and then rehydrated with xylene and aqueous ethanol. Incubation with 3% hydrogen peroxide for 15 minutes to block endogenous peroxidase activity. The slices immersed in 10 mmol/L citric acid solution (PH6) were microwave at high-power for 7 minutes, and then washed with phosphate buffered saline (PBS) for 3 minutes. The sections were then incubated with antibody with blocking solution overnight at 4°C. Then three PBS washes were performed, after which the slides were exposed to the secondary antibody for 1 h at room temperature. Wash 3 times with PBS, 5 minutes each time, DAB color development for 5 minutes, tap water for 10 minutes, hematoxylin counterstain for 2 minutes, hydrochloric acid alcohol differentiation, tap water for 10 minutes, then routine dehydration, transparency, mounting, and microscopy.

**qRT-PCR, RT-PCR, and western blotting**

Trizol reagent (Invitgen, USA) was used for extraction and isolation of total RNA. Reverse transcription of 500 ng of total RNA was performed using PrimeScript PT kit (Kogen, Japan). The mRNA expression level was detected by qRT-PCR using qPCR SYBR Green Master Mix from YEASEN (11202ES03). The PCR amplification procedure was performed following the instructions of the reagents. ACTB and 18S were used as endogeneous controls to normalize the RNA content in cells and tissues, respectively.

RT-PCR were performed by using the AceTaq Master Mix (P412-01) from Vazyme for amplification. Prepare 1~2% agarose gel solution, add 5ul 10,000* Ultra GelRed nucleic acid dye to 50ml agarose gel solution. Put the gel in the electrophoresis tank, remove the comb, add 1*TAE electrophoresis buffer to 1~2mm above the gel plate, add the 100bp marker to the slots on both sides, and add the samples according to the order. After electrophoresis at 120V for 30 minutes, the gel was taken out, observed under an ultraviolet light, photographed and saved using the gel imaging system.

For western blotting, under the microscope, when the cell reached 90% confluence, wash 3 times with pre-cooled PBS, and then extract the total proteins in RIPA buffer containing phosphatase and protease inhibitors. Extract the total protein based on the instructions. Add 5*SDS loading buffer to all samples, and put them in a metal bath at 100°C for 5 minutes. The extracted protein (50μg) was separated by 10% sodium dodecyl sulfate-polyacrylamide gel electrophoresis (SDS-PAGE). Transfer the extracted sample to a polyvinylidene difluoride membrane (MilliPore, USA), which was blocked by incubation at room temperature for 1h with TBST buffer (20 mMTris/HCl, pH 7.5; 0.137M NaCI; 0.05% Tween-20) containing 5% bovine serum albumin (BSA). Then use the primary antibodies diluent to dilute the antibodies according to the instruction of the antibodies, and incubate overnight at 4°C. Wash 3 times with TBST, 10 minutes each time. Then the secondary antibodies diluted with TBST were added to incubate at room temperature for 1 hour, Wash 3 times with TBST, 10 minutes each time. Detect proteins with enhanced chemiluminescence method. GAPDH and β-tubulin were used as loading controls. Each experiment was performed three times.

**Motif analysis of SRSF1-mediated alternative exons**

Firstly, enrichments of GA-rich regions were identified by counting the occurrences of each eligible 6-mer within five segments of each transcript with a SRSF1-regulated cassette exon (including the cassette exon and the two flanking constitutive exons and introns). The cassette exons, which were not affected by SRSF1, were treated as the control group. Secondly, conduct a hypergeometric test (Fisher’s right-tailed exact test) to detect the enrichment of each hexamer sequence within the sequences undergoing SRSF1-regulated inclusion (or exclusion) events compared to the control sequences. A Bonferroni-adjusted P-value (or false discovery rate, FDR) was performed by multiplying the actual total number of tests. Next, calculate the sums of the minus log2-transformed P-values for GA-rich 6-mers as a metric to determine the peaks of GA-rich 6-mers within the five regions around the regulated cassette exons. Among the identified GA-rich peaks, the overrepresented GA-rich 6-mers (P<0.0001) along with flanking 5 nt on each side (a total of 16 nt) were further collected and submitted to MEME suite for possible GA-rich motif analysis. The parameter setting for MEME was a number of single motif per sequence of 0 or 1, a minimum motif width of 5, a maximum motif width of 8, a maximum number of different motifs of 3 and a search of only the given strand. Finally, MEME searching motif enrichments were manually edited to make it a 6-mer length.

**Minigene reporter assay**

1. Construction and verification of PTPMT1-FL

There are 4 exons in PTPMT1, among which exon 3 can undergo abnormal AS splicing. First, construct a PTPMT1 full length (PTPMT1-full length, PTPMT1-FL) plasmid that can mimic PTPMT1 pre-RNA in vitro, this plasmid was ordered from Beijing Kinco Biotechnology Co., Ltd. The empty vector is pcDNA3.1, and the insert is sequentially exon 2, 300bp at each end of intron 2, exon 3, 300bp at each end of intron 3, exon 4. Inoculate 293T into a 6-well plate, and transfect the PTPMT1-FL, PTPMT1-FL+shSRSF1, PTPMT1-FL+SRSF1 overexpression plasmids into the three wells. After culturing 48h, collect the cells for RT-PCR to verify that PTPMT1-FL can mimic the regulation of PTPMT1 pre-RNA AS by SRSF1 in vivo.

2. Construction of deleted mutant plasmid

1) According to literature reports and sequencing results, two deletion sequences were determined respectively (E3F1: GAAGAGACTAGGAG; E3F2: CCAGTCGCTGGGCCAGTGTGTTTACGTGCATTGTAAGGCTGGGCGCTCCAGGAG);

2) Design primer: forward primer: put 15bp on both sides of the deleted fragment, and end with G/C on the 3'end (strong binding force). The reverse primer is the reverse complementary strand of the forward primer. Synthesize the primer.

3) Prepare PCR reaction system (50ul) according to the following table:

| reagents | quantity |
| --- | --- |
| 10*buffer（for KOD plus） | 5ul |
| MgSO4 | 2ul |
| dNTP  Forward primer(3uM)  Reverse primer(3uM)  DNA template（PTPMT1-FL plasmids）  KOD enzyme  ddH2O | 5~8ul  4ul  4ul  5~10ng  1ul  Add to 50ul |

4) Set the PCR procedure based on the following table:

| temperature | time | cycle |
| --- | --- | --- |
| 95℃ | 8min |  |
| 95℃ | 30s |  |
| 50℃ | 50s | 5 cycles |
| 68℃ | 8min* |  |
| 95℃ | 30s |  |
| 55℃ | 50s | 18cycles |
| 68℃ | 8min |  |
| 68℃ | 8min |  |
| 12℃ | ∞ |  |

*：Adjust the time according to the size of the fragment (1min/1kb)

5) Take 5ul product and run 1% nucleic acid agarose gel. According to the instructions of the marker, if there is a band in the corresponding size position, continue with the following operations;

6) Remove the original plasmid: add 5ul cut smart buffer, 1ul DpnI to the remaining 45ul product, put it in a 37℃ water bath for 1~2h after mixing;

7) Transformation: TSC01 Super Competent was purchased from Beijing Kinco Biological Co., Ltd. Take 100ul of competent cells melted on ice, add 10ul of reaction product, mix gently, and let stand on ice for 30min. Heat shock in 42℃ water bath for 45s, quickly transfer to ice bath, and let stand for 2min. Add 500ul of non-resistant LB broth to the centrifuge tube and resuscitate at 37°C/200rpm for 60 minutes. Take a suitable volume of the resuscitation solution and evenly spread it on the medium containing the corresponding antibiotics, and invert it in the 37°C incubator overnight.

8) Picking clones and sequencing identification: The next day, gently pick out the monoclonal colonies with a sterilized white pipette tip, inoculate them into a 15ml centrifuge tube and add 5ml medium containing the corresponding antibiotics, 200rpm, 37℃ shaking for 6~8h. Take 300~500ul of bacterial solution and send it to the company for sequencing, and store the rest in a refrigerator at 4℃. Expand the bacterial solution with correct sequencing, extract the plasmid according to the instructions of the plasmid extraction kit, and store it at -20°C for later use.
